# Stroke-prone salt-sensitive spontaneously hypertensive rats show higher susceptibility to spreading depolarization (SD) and altered hemodynamic responses to SD

**DOI:** 10.1177/0271678X221135085

**Published:** 2022-11-03

**Authors:** Eun-Jeung Kang, Ofer Prager, Svetlana Lublinsky, Ana I Oliveira-Ferreira, Clemens Reiffurth, Sebastian Major, Dominik N Müller, Alon Friedman, Jens P Dreier

**Affiliations:** 1 Center for Stroke Research Berlin, Charité – Universitätsmedizin Berlin, Corporate Member of Freie Universität Berlin, Humboldt-Universität zu Berlin, and Berlin Institute of Health, Berlin, Germany; 2 Department of Experimental Neurology, Charité – Universitätsmedizin Berlin, Corporate Member of Freie Universität Berlin, Humboldt-Universität zu Berlin, and Berlin Institute of Health, Berlin, Germany; 3 Department of Physiology & Cell Biology, The Zlotowski Center for Neuroscience, Ben-Gurion University of the Negev, Beer-Sheva, Israel; 4 Department of Cognitive & Brain Sciences, The Zlotowski Center for Neuroscience, Ben-Gurion University of the Negev, Beer-Sheva, Israel; 5 Department of Neurology, Charité – Universitätsmedizin Berlin, Corporate Member of Freie Universität Berlin, Humboldt-Universität zu Berlin, and Berlin Institute of Health, Berlin, Germany; 6 Experimental and Clinical Research Center (ECRC), a Joint Cooperation between the Charité – Universitätsmedizin Berlin and Max-Delbrück-Center for Molecular Medicine, Berlin, Germany; 7 Max-Delbrück-Center for Molecular Medicine in the Helmholtz Association (MDC), Berlin, Germany; 8 Department of Medical Neuroscience and Brain Repair Center, Dalhousie University, Halifax, Nova Scotia, Canada; 9 Bernstein Center for Computational Neuroscience Berlin, Berlin, Germany; 10 Einstein Center for Neurosciences Berlin, Berlin, Germany

**Keywords:** Spreading depolarization, spreading depression, salt-sensitivity, hypertension, stroke, migraine

## Abstract

Spreading depolarization (SD) occurs in a plethora of clinical conditions including migraine aura, delayed ischemia after subarachnoid hemorrhage and malignant hemispheric stroke. It describes waves of near-breakdown of ion homeostasis, particularly Na^+^ homeostasis in brain gray matter. SD induces tone alterations in resistance vessels, causing either hyperperfusion in healthy tissue; or hypoperfusion (inverse hemodynamic response = spreading ischemia) in tissue at risk. Observations from mice with genetic dysfunction of the ATP1A2-encoded α_2_-isoform of Na^+^/K^+^-ATPase (α_2_NaKA) suggest a mechanistic link between (1) SD, (2) vascular dysfunction, and (3) salt-sensitive hypertension via α_2_NaKA. Thus, α_2_NaKA-dysfunctional mice are more susceptible to SD and show a shift toward more inverse hemodynamic responses. α_2_NaKA-dysfunctional patients suffer from familial hemiplegic migraine type 2, a Mendelian model disease of SD. α_2_NaKA-dysfunctional mice are also a genetic model of salt-sensitive hypertension. To determine whether SD thresholds and hemodynamic responses are also altered in other genetic models of salt-sensitive hypertension, we examined these variables in stroke-prone spontaneously hypertensive rats (SHRsp). Compared with Wistar Kyoto control rats, we found in SHRsp that electrical SD threshold was significantly reduced, propagation speed was increased, and inverse hemodynamic responses were prolonged. These results may have relevance to both migraine with aura and stroke.

## Introduction

The neurovascular unit (NVU) is comprised of vascular cells [endothelium, vascular smooth muscle cells, pericytes], glia (astrocytes, microglia, oligodendrocytes), and neurons. For a long time, there was a clear division of tasks between neurons on the one hand, which perform the core function of the brain, namely information processing, and the remaining cell types on the other hand, which feed the neurons, ensure the removal of metabolic waste products, for these purposes form a barrier between circulation and parenchyma (the blood-brain barrier (BBB)), match regional cerebral blood flow (rCBF) to neuronal energy demand and perform immunological surveillance. In recent decades, however, it has become increasingly clear that this view is too simplistic and that, for example, astrocytes serve important roles in information processing as they affect recruitment and function of neurons at local and network levels.^1^ Similarly, only the other way round, the dogma of stroke was for a long time that it is always a primarily vascular disorder in which neurons and astrocytes are only passively involved. However, in the past 25 years, even this view has fundamentally changed, as it has been discovered that primary neuronal disruptions of ion homeostasis, so-called spreading depolarizations (SD), which initiate and maintain neuronal cytotoxic edema,^2–4^ can secondarily trigger severe vasoconstriction and spreading ischemia, leaving neurons trapped in the disrupted state that eventually leads to ischemic necrosis.^5,6^ SD is observed as a large negative direct current (DC) shift and associated with a near-complete breakdown of transmembrane ion gradients and neuronal swelling.^7^ In alternating current (AC) electrocorticography (ECoG), SD triggers a rapidly evolving reduction in the amplitudes of spontaneous activity, termed spreading depression.^8^ SD-induced spreading ischemia has become the best characterized stroke mechanism in humans in terms of the pathophysiological relationships between DC/AC-ECoG, rCBF, and tissue partial pressure of oxygen (p_ti_O_2_), as it typically occurs in patients during neurocritical care in whom neurosurgical intervention is indicated, allowing implantation of invasive probes for advanced neuro- and vascular monitoring before, during, and after the development of ischemic infarcts. In particular, SD-induced spreading ischemia is a key process in the pathogenesis of early and delayed infarcts in patients after aneurysmal subarachnoid hemorrhage (aSAH).^9–13^ Furthermore, spreading ischemia was found in patients with traumatic brain injury (TBI)^14^ and exacerbated preexisting ischemia in malignant hemispheric stroke (MHS) after middle cerebral artery (MCA) occlusion (MCAO).^15^

SD induces tone alterations in resistance vessels, causing either predominant hyperperfusion followed by a mild oligemia (physiological hemodynamic response) in healthy tissue;^16–18^ or severe and prolonged initial hypoperfusion (inverse hemodynamic response = spreading ischemia) when the NVU is severely disturbed.^5,6^ Spreading ischemia is distinguished from primary ischemia, such as occurs in the setting of embolic/thrombotic occlusion of a major cerebral artery. Whereas in the case of spreading ischemia, SD occurs first and is followed by ischemia with a latency of several seconds, and both SD and ischemia spread in the tissue,^5,6^ in the case of severe primary ischemia, ischemia occurs first, followed by SD with a substantial latency of ∼1–5 minutes, and only SD, but not ischemia, spreads in the tissue.^10,19,20^ Nevertheless, the process of spreading ischemia can also build up on incomplete primary ischemia,^21–23^ as is the case, for example, in the context of photothrombosis in mice.^24^ In this model, the initial ischemia as a direct consequence of photothrombosis is relatively mild. Only when the first SD occurs between 3 and 35 minutes after the onset of photothrombosis does it result in severe spreading ischemia that produces an ischemic core and becomes progressively milder as it migrates out of the photothrombotic zone. In addition, even if the ischemic core is already permanently depolarized, severe, sporadic SD-induced spreading ischemia may still occur in the penumbra several hours after MCAO, for example, in cats, resulting in delayed growth of the core.^22^ Such severe, sporadic SD-induced spreading ischemia in penumbral tissue also occurs in rats, including spontaneously hypertensive rats (SHR), and in patients.^15,23,25^

In spreading ischemia, the rCBF decline leads to a repercussion on the negative DC shift which becomes longer-lasting compared to the one in normal tissue. This results from mismatch between energy demand and supply, which causes insufficiency of membrane pumps to repolarize the neurons.^6^ In the setting of short-duration SD and normal hemodynamic response, SD does not cause neuronal damage locally.^26^ However, spreading ischemia can lead to infarction even in tissue that was not yet ischemic at the onset of SD.^27^ Importantly, SD properties can change continuously and massively depending on local conditions as an SD moves from one site to another through the tissue. Overall, rather than the number of SDs, their local duration, measured as the local duration of the negative DC shift and depending in particular on both baseline rCBF and hemodynamic response, indicates the local risk of cell death development.^7,28,29^ In this concept, baseline rCBF and hemodynamic response are complex variables, through which the local duration of SD and neuronal cytotoxic edema, and thus the local risk of cell death development can be modulated, for example, by preconditioning^30^ or pharmacological treatment.^6^

Furthermore, a relevant aspect is that although SD begins in neurons and other cell types of the NVU are only secondarily involved,^31–34^ the dysfunction causative for the initiation of SD in neurons may well be of astrocytic origin.^35^ This has been particularly well evidenced, for example, for familial hemiplegic migraine type 2 (FHM2), a rare Mendelian model disease of SD due to loss-of-function mutations in ATP1A2, the gene encoding the α_2_ isoform of Na^+^/K^+^-ATPase (NaKA).^36–41^

Another disease that has long been considered primarily vascular and is now increasingly thought to involve neuronal and astrocytic brain dysfunction causally in its pathogenesis is salt-sensitive hypertension. In patients, salt-sensitivity is present when the blood pressure changes by 5% to 10% or at least 5 mmHg, in response to increased NaCl intake.^42^ Fifty-one percent of the hypertensive and 26% of the normotensive population are salt-sensitive.^43^ Animal studies suggest that genetic predisposition has a significant influence on whether or not an individual responds to NaCl intake with hypertension.^42^ Thus, polygenic animal models such as SHR,^44^ SHR-derived stroke-prone SHR (SHRsp)^45^ and Dahl salt-sensitive rats were bred, which are characterized by a particularly high salt-sensitivity.^46–49^ Dysfunctions in two organs in particular, namely kidney and brain, are held responsible for salt-sensitivity. At the level of the brain, the aldosterone-epithelial Na^+^ channel (ENaC)-endogenous ouabain-angiotensin II receptor type 1 (AT1R) cascade has been implicated as the causative factor in this common disorder.^50^ This cascade involves three hormone-receptor pairs: (1) aldosterone and mineralocorticoid receptors, (2) endogenous ouabain and α_2_NaKA, and (3) angiotensin II and AT1R.^50^ It is assumed that a set-point adjustment in this neural circuitry causes increased salt-sensitivity. Interestingly, at the center of this set-point adjustment is not a change in a neuronal hormone receptor but in an astrocytic/vascular hormone receptor, namely α_2_NaKA.^50–52^ According to this concept, salt-sensitive hypertension is a model disease in which hormonal astrocytic/vascular changes unbalance a neural circuitry, which in turn leads to a complex systemic vascular disorder.^50,52^ However, genetic and pharmacological reductions in α_2_NaKA-activity not only lead to salt-sensitivity,^51,53–55^ but also to a higher susceptibility to SD^37–41,56^ and a higher propensity to react to SD with inverse hemodynamic responses.^40,56^ This commonality suggests that considering all three conditions together may yield new insights.

The very first question that arises here is whether genetic predisposition to salt-sensitivity in general, i.e., in patients with salt-sensitive hypertension or in other experimental models than α_2_NaKA-dysfunctional mice, is associated with increased susceptibility to SD and increased propensity to react to SD with inverse hemodynamic responses. SHRsp, along with α_2_NaKA-dysfunctional mice, are another important genetic model for salt-ensitivity, thought to be due to a dysfunctional aldosterone-ENaC-endogenous ouabain-AT1R cascade in the brain.^46–50,57^ Although the ATP1A2 gene indeed shows characteristic differences in SHRsp compared with normotensive Wistar-Kyoto (WKY) or Sprague-Dawley rats^58^ and SHRsp exhibit greatly enhanced sensitivity to ouabain,^57,59^ the mechanisms underlying the dysfunctionality of the aldosterone-ENaC-endogenous ouabain-AT1R cascade may be more complicated in SHRsp than α_2_NaKA-dysfunctional mice. However, to explore this further was not the purpose of the present work; rather, we performed here a deep phenotyping of SHRsp with respect to SD and the inverse hemodynamic response because we considered this the first logical step to determine whether there might be a fundamental association between the genetic predispositions to salt-sensitivity, SD, and inverse response beyond α_2_NaKA-dysfunctional mice.

SHRsp develop progressive hypertension during young adulthood. The phenotype is polygenic and associated with over-activity of the renin-angiotensin-aldosterone system (RAAS). Histologic studies of small penetrating arteries showed several features of fibrinoid vasculopathy in common with human small vessel disease.^60^ SHRsp are regarded as a model of endothelial dysfunction.^61^ The incidence of ischemic strokes is rare when SHRsp are 12–14 weeks old, but exceeds 80% by 30 weeks. The onset of cerebrovascular lesions in SHRsp is dramatically accelerated by exposure of the rats to the so-called Japanese dietary regimen based on a high Na^+^ intake, high Na^+^/K^+^ ratio, and a modified protein composition.^49,62,63^ This enhancement of the SHRsp phenotype is more complex than pure salt-sensitivity, because the lower-protein diet accelerated stroke development for the same salt load, although blood pressure tended to be somewhat lower.^63^

To ensure that our results are not directly influenced by previous strokes, we studied 12–14-week-old SHRsp compared with WKY control rats. In *in vivo* experiments, we compared the electrical SD threshold, SD propagation speed, normal hemodynamic response to SD and post-SD edema formation between SHRsp and WKY on regular diet. In another series, spreading ischemia was induced by topical application of artificial cerebrospinal fluid (aCSF) on the brain containing the nitric oxide synthase (NOS) inhibitor N^G^-nitro-L-arginine (L-NNA) and increased K^+^ concentration ([K^+^]_aCSF_) as reported previously.^5,12,56,64^ Spreading ischemia was compared between four groups: SHRsp and WKY on Japanese diet and on regular diet.

## Materials and methods

### Animals

The reporting complies with the ARRIVE Guidelines. All animal experiments were authorized by the animal welfare authorities in Berlin, Germany: Berlin State Office for Health and Social Affairs (LAGeSo), G0203/13, and all experimental procedures were conducted in accordance with the Charité Animal Welfare Guidelines. The animals were housed in groups (two animals/cage) under a 12 h light/dark cycle with food and tap water available ad libitum.

**Figure 1.**
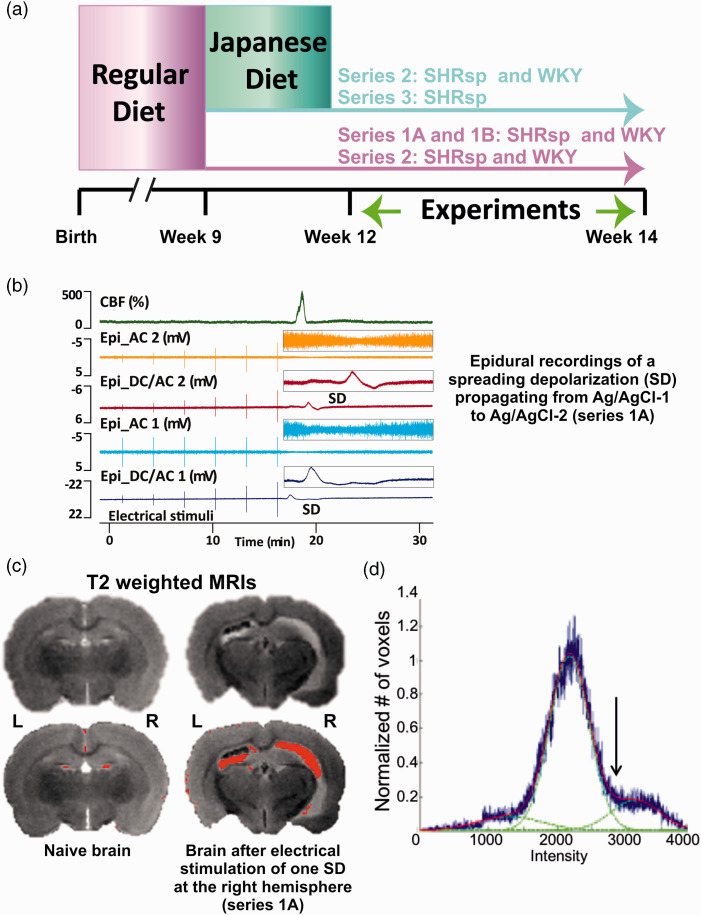
Experimental set-ups and protocols. (a) In the first 9 weeks, all animals received a regular diet. After that, part of the animals continued to receive regular diet and another part was fed with Japanese diet. The experiments were performed in 12 to 14 weeks old animals. (b) Original recording of a spreading depolarization (SD) in series 1A. Trace 1 from top to bottom shows the normal hyperemic regional cerebral blood flow (rCBF) response to SD. Traces 2 and 4 demonstrate the SD-induced spreading depression of activity in the alternating current (AC)-frequency band and traces 3 and 5 the negative direct current (DC) shift of SD at the two epidural recording sites. Vertical lines are electrical stimulation artifacts. The insets on the right give the DC/AC-electrocorticography (ECoG) changes of the SD on an expanded time scale and y axis. The SD propagates from the rostral to the caudal epidural recording site (Epi_DC/AC 1 to Epi_DC/AC 2). (c) Post-mortem magnetic resonance imaging (MRI) using a T2-weighted (T2w) fast spin echo (FSE) protocol of a naïve stroke-prone spontaneously hypertensive rat (SHRsp) and an SHRsp after stimulation of one SD (right hemisphere) (series 1A). High signal intensity (bottom – overlaid in red) suggests either cerebrospinal fluid (CSF) or vasogenic edema.^74^ and (d) Voxel-based analysis of variability in T2w image intensity to identify edema. A Gaussian mixture model with three Gaussian probability density functions was applied to model the variety of intensities. Accordingly, voxels were clustered as a mixture of three Gaussians, namely: “low”, white matter; “medium”, mostly gray matter; and “high”, ventricles or abnormally hyperintense regions. Hyperintensities were quantified by including voxels above a segmentation threshold, defined as intersection between 2nd and 3rd Gaussian fit (arrow).

In the first 9 weeks, all animals received a regular diet. In series 1A and 1B, both SHRsp and WKY rats continued to receive regular diet as shown in Figure 1(a). In contrast, in series 2, both SHRsp and WKY rats were divided into two subgroups each after the first 9 weeks. One received normal rat chow (containing 22% protein, 2.7 mg/g Na^+^, 7.4 mg/g K^+^, 0.05 mg/g methionine) and tap water, the other received a high-salt, mildly reduced protein Japanese diet [containing 17.5% protein, 3.7 mg/g K^+^, and 0.03 mg/g methionine (ssniff Spezialdiäten GmbH, Soest, Germany, Supplementary Table 1)] and 1% NaCl added to the drinking water.^65^ Series 3 only contained SHRsp rats that received Japanese diet after the first 9 weeks. All experiments were performed when the animals were 12–14 weeks old. Male SHRsp rats (n = 59) and male WKY rats (n = 46) were anesthetized with 100 mg/kg body weight (BW) thiopental sodium intraperitoneally (Trapanal®, BYK Pharmaceuticals, Konstanz, Germany), tracheotomized and artificially ventilated (Effenberger Rodent Respirator; Effenberger Med.-Techn. Gerätebau, Pfaffing/Attel, Germany) to maintain an arterial partial pressure of CO_2_ (pCO_2_) between 35 and 45 mmHg, an arterial pO_2_ between 90 and 130 mmHg and an arterial pH between 7.35 and 7.45. The right femoral artery and vein were cannulated and saline solution was continuously infused to keep the vessels open (1 ml/h). Systemic mean arterial pressure (MAP, Pressure Monitor BP-1, World Precision Instruments, Berlin, Germany) and expiratory pCO_2_ (Heyer CO_2_ Monitor EGM I, Bad Ems, Germany) were continuously monitored. Arterial pO_2_, pCO_2_ and pH were serially measured (AVL Medizintechnik GmbH, Bad Homburg, Germany). A physiological body temperature was maintained using a rectal probe connected to a heating pad (Homeothermic Blanket Control, Harvard Apparatus, Cambridge, MA, U.S.A.). Anesthesia was assessed by testing motor responses and changes in MAP to foot-pinching. If necessary, additional doses of thiopental (25 mg/kg BW) were applied.

### Cranial window preparation and experimental set-up

**Figure 2.**
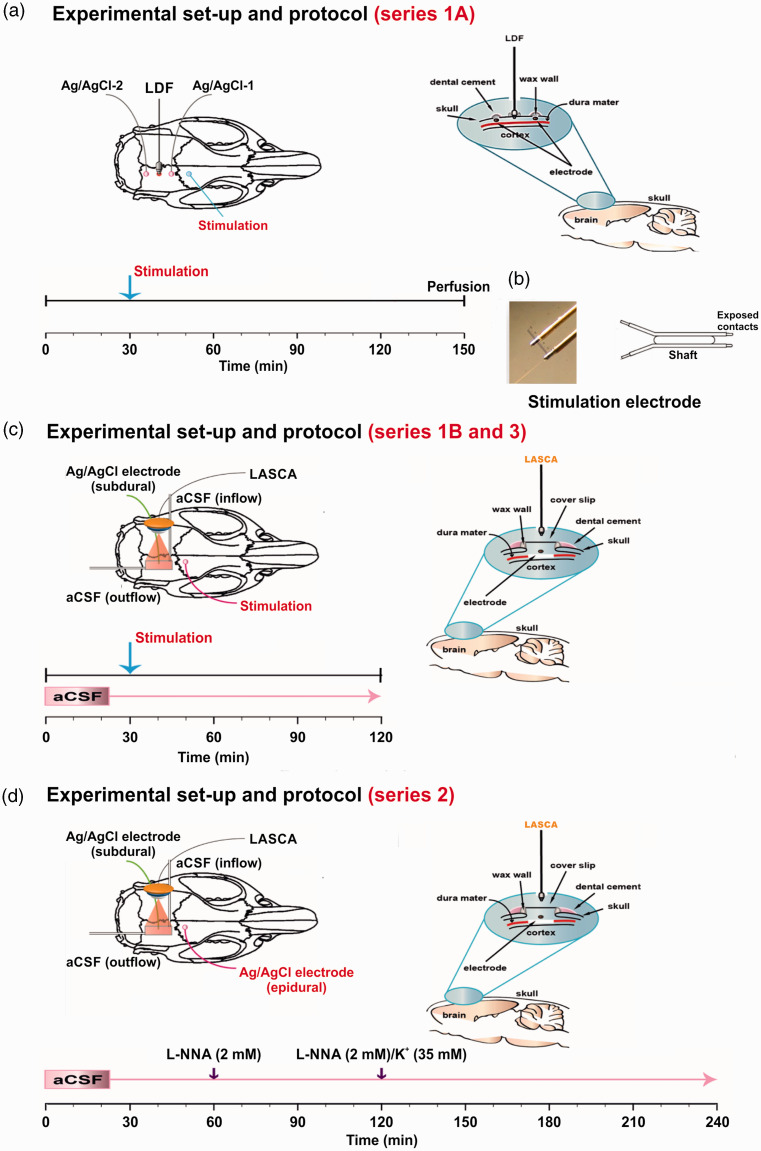
(a) Experimental set-up of series 1A. To determine the threshold of spreading depolarization (SD), two silver/silver chloride (Ag/AgCl) electrodes were inserted over the right parietal cortex which measured the epidural direct current (DC)/alternating current (AC)-electrocorticogram (ECoG). Another burr hole was implanted in-between to measure the corresponding regional cerebral blood flow (rCBF) changes using a laser-Doppler flowmetry (LDF) probe. SDs were elicited by current pulses of increasing intensity using a frontal bipolar stimulation electrode. The right panel shows the burr holes for epidural recordings and the lower panel the experimental protocol of series 1A. (b) Bipolar stimulation electrode (2 × 0.2 mm insulated stainless steel wires separated by a 0.5 mm shaft). (c) The left panel shows the experimental set-up of series 1B and 3. aCSF: artificial cerebrospinal fluid, LASCA: laser speckle contrast analysis imaging (sampling rate: 0.5 Hz, spatial resolution: 20 µm, averaged frames per image: 10). The right panel shows the closed cranial window used in series 1B and 3 and the lower panel the experimental protocol. (d) The left panel shows the experimental set-up, the right panel the closed cranial window and the lower panel the experimental protocol of series 2.

Figure 2 illustrates the experimental set-ups and paradigms. In series 1A, one rostral and one caudal burr hole (diameter: ∼1.5 mm; 2–3 mm lateral to midline; ∼1.5 mm and ∼6.5 mm caudal to bregma) were placed over the parietal cortex for epidural recordings with two Ag/AgCl electrodes. A third burr hole for a laser-Doppler flow (LDF) probe (Periflux 4001, Perimed, Järfälla, Sweden) was implanted between the two electrodes to measure rCBF as reported previously (Figure 2(a)).^66,67^ A fourth burr hole was drilled over the frontal cortex at a distance of 4 mm to the rostral burr hole for placement of a bipolar stimulation electrode (NE-200, Rhodes Medical Instruments, Summerland, CA, USA) (Figure 2(b)).

In series 1B and 3 (Figure 2(c)), and series 2 (Figure 2(d)), a craniotomy (4x5mm) was performed over the parietal cortex, the dura was removed, and a closed cranial window was implanted as reported previously.^68^ The ECoG was subdurally recorded at the closed window using an Ag/AgCl electrode inserted into the space between cortex and coverslip. Laser speckle contrast analysis (LASCA) imaging was employed to map cerebral perfusion levels in the window area as reported previously (PeriCam PSI HR, Perimed Instruments, Järfälla, Sweden, analysis with PIMSoft, Perimed Instruments).^40^ The cortical surface at the cranial window was continuously superfused with aCSF containing in mM: 127.5 NaCl, 24.5 NaHCO_3_, 6.7 urea, 3.7 glucose, 3 KCl, 1.5 CaCl_2_, and 1.2 MgCl_2_. In series 2, the Na^+^ concentration in aCSF ([Na^+^]_aCSF_) was simultaneously decreased from 152 to 120 mM to keep osmolarity constant when [K^+^]_aCSF_ was increased from 3 to 35 mM to induce spreading ischemia. The aCSF was equilibrated with a gas mixture containing 6% O_2_, 5.9% CO_2_, and 87.5% N_2_ to achieve physiological levels of pO_2_, pCO_2_ and pH. An additional burr hole was implanted rostrally to either place a bipolar stimulation electrode (series 1B and 3) (Figure 2(c)) or to perform epidural recordings with an Ag/AgCl electrode (series 2) (Figure 2(d)).

### Electrical stimulation of SD

See Supplementary Materials and Methods.

### Animal recording techniques

Subdural and epidural DC/AC-ECoG was performed to monitor SDs and SD-induced activity depression as reported previously.^7,68^ Electrodes were connected to a differential amplifier (Jens Meyer, Munich, Germany). Analog-to-digital conversion was performed using a Power 1401 (Cambridge Electronic Design Limited, Cambridge, UK). Relative rCBF changes were calculated in relation to baseline after a stabilization period (=100%). If possible, a zero level was established at the end of the experiment after cardiac arrest by air embolism. Regions of interest (ROI) in LASCA imaging covered an area of 0.2 mm^2^. MAP, expiratory pCO_2_, DC/AC-ECoG and rCBF (LDF) were continuously recorded using a personal computer and Spike 2 software (version 6, Cambridge Electronic Design Limited, Cambridge, UK).

### Experimental paradigms

In series 1A, we first determined the electrical threshold of SD in SHRsp and WKY rats. SD propagation speed was calculated from the delay between the SD-initiating electrical stimulus and SD onset at the rostral window and the distance between stimulation site and rostral window. After recovery from SD, intracardial perfusion of phosphate-buffered saline (PBS) containing 4% paraformaldehyde (PFA) was performed (Figure 2(a)). Brains were removed and fixed for 48 hours in 4% PFA, and were then transferred to store-solution (0.25% PFA in PBS) for *ex vivo* magnetic resonance imaging (MRI).

In series 1B and 3, normal aCSF was brain topically applied throughout the experiment (Figure 2(c)). We determined the electrical threshold of SD and recorded the normal hemodynamic response in the window area with LASCA imaging. In order to determine the SD propagation speed in these experiments, a rostral and a caudal ROI were defined. When SD was electrically triggered, this was detected as a hyperemic wavefront in the window area that propagated from rostral to caudal. SD propagation speed was calculated from the delay between the appearance of the hyperemic wavefront at the rostral and caudal ROI and the distance between the two ROIs. Series 3 was performed after the other series were completed. In contrast to series 1B, in which SHRsp and WKY received regular diet, series 3 consisted of only SHRsp on Japanese diet. The reason for our decision to perform an additional series 3 after we had completed the data analysis from series 1 and 2 was to exclude the possibility that a Japanese diet alone, i.e. without concomitant topical application of aCSF containing L-NNA and increased [K^+^]_aCSF_, was sufficient to cause SD-induced spreading ischemia in SHRsp.

In series 2, the standard pharmacological protocol for eliciting the inverse hemodynamic response to SD was used (Figure 2(d)).^5^ Thus, after an equilibration period of 1 hour, aCSF containing L-NNA at 2 mM was applied brain topically for 1 hour. Thereafter, aCSF containing L-NNA at 2 mM and elevated [K^+^]_aCSF_ at 35 mM instead of 3 mM was brain topically applied for 2 hours to elicit SD and SD-induced spreading ischemia. The SD propagation velocity was calculated similarly to series 1B and 3. However, the delay between the appearance of the hypoemic wavefront at two different ROIs was used for this purpose.

### Ex-vivo MRI

See Supplementary Materials and Methods.

### Experimental design and statistical analysis

See Supplementary Materials and Methods.

## Results

### BW before surgery, and body temperature and MAP after surgery

**Figure 3.**
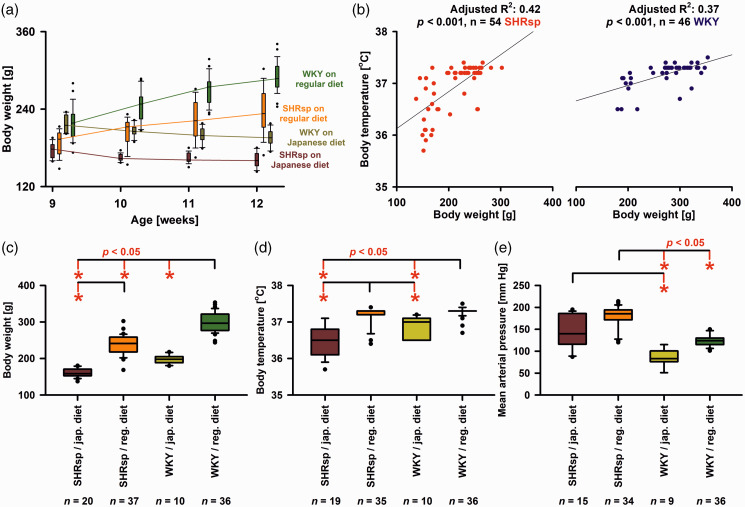
Body weight (BW), body temperature and mean arterial pressure (MAP) in stroke-prone spontaneously hypertensive rats (SHRsp) and Wistar-Kyoto rats (WKY) on either regular or Japanese diet. (a) Evolution of BW in SHRsp and WKY on regular or Japanese diet at weeks 9, 10, 11, and 12 before experiments conducted at weeks 12-14. For the statistical analysis, we used a two-way repeated-measures analysis of variance (ANOVA) on ranks with post-hoc Bonferroni t-tests [SHRsp on Japanese diet (*n* = 19) versus SHRsp on regular diet (*n* = 16) versus WKY on Japanse diet (*n* = 11) versus WKY on regular diet (*n* = 28) as factor A and age in weeks as factor B]. As early as week 9 before starting the Japanese diet in two groups, BW was significantly higher in WKY than SHRsp (*p* ≤ 0.05). At week 12, all 4 groups were significantly different from each other (*p* ≤ 0.05). Within all 4 groups, there was a significant difference between BW at week 9 and week 12 (*p* ≤ 0.05). That is, there was significant weight loss in both strains under Japanese diet and significant weight gain under regular diet, which suggests some degree of malnutrition under Japanese diet. (b) When experiments were pooled linear regression found that BW immediately before the experiments and body temperature after surgery significantly correlated both in SHRsp and WKY. The relationship between body weight and body temperature observed here is related to the so-called Bergmann's rule. Thus, as the volume of an object decreases, the ratio of its surface area to its volume increases. In other words, the smaller an animal is, the higher the surface area-to-volume ratio. These animals lose heat relatively quickly and cool down faster. (c) Immediately before the experiments at the age of 12–14 weeks, WKY rats on regular diet had a significantly higher median BW than WKY on Japanese diet, SHRsp on regular diet, or SHRsp on Japanese diet [Kruskal-Wallis One Way Analysis of Variance on Ranks (KW-ANOVA) and post-hoc Dunn’s tests (phD)]. In addition, SHRsp on regular diet had a significantly higher median BW than SHRsp on Japanese diet. (d) Body temperature before the start of the experiments was significantly higher in WKY on regular diet than in either WKY on Japanese diet or SHRsp on Japanese diet (KW-ANOVA and phD). In addition, body temperature was significantly higher in SHRsp on regular diet than in either WKY or SHRsp on Japanese diet. SD susceptibility is known to correlate positively with body temperature.^136^ Because the body temperature of SHRsp tended to be lower than that of WKY, it can be excluded that changes in body temperature were responsible for the higher SD susceptibility of SHRsp and (e) MAP before the start of experiments was significantly higher in SHRsp on regular diet than in WKY on either regular diet or Japanese diet (KW-ANOVA and phD). In addition, MAP was significantly higher in SHRsp on Japanese diet than WKY on Japanese diet. The whiskers (error bars) above and below the boxes indicate the 90th and 10th percentiles.

Figure 3 summarizes the effects of rat strain and diet type on BW development before the experiments, as well as on body temperature and MAP measured immediately after surgery. In essence, the results show the expected differences between the four groups. In addition, when experiments were pooled BW immediately before the experiments and body temperature after surgery significantly correlated both in SHRsp and WKY (Figure 3(b)).

### Electrical threshold and propagation speed of SD in SHRsp and WKY rats

**Figure 4.**
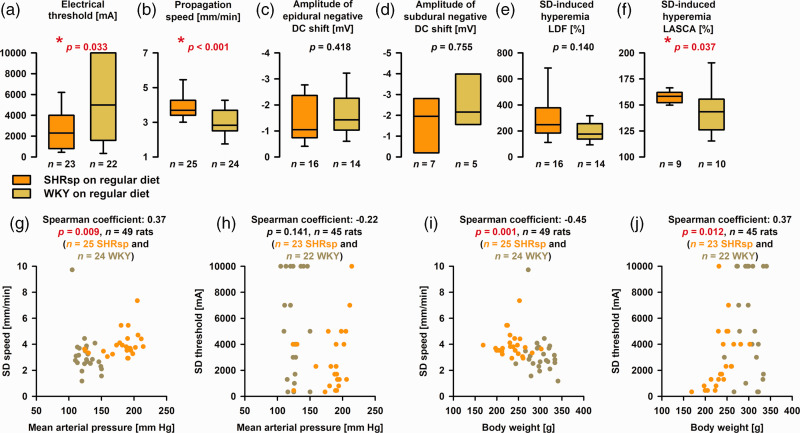
Differences in spreading depolarization (SD) threshold and propagation speed between stroke-prone spontaneously hypertensive rats (SHRsp) and Wistar-Kyoto rats (WKY) on regular diet. (a) We found that the median electrical threshold of SD was significantly lower in SHRsp than WKY [Mann-Whitney U test (MWU)]. (b) The median propagation speed of SD was significantly faster in SHRsp than WKY (MWU). (c) In series 1A, the amplitude of the epidural negative DC shift was not different between SHRsp and WKY (MWU). (d) In series 1B, the amplitude of the subdural negative DC shift in the window area was not different between SHRsp and WKY (MWU). (e) In series 1A, the peak of the laser-Doppler flowmetry (LDF)-determined hyperemia was not significantly different between SHRsp and WKY (MWU). (f) In series 1B, we determined the median of 5 regions of interest (ROI) in the window area for each animal. We found that the laser speckle contrast analysis (LASCA) imaging-determined hyperemia was significantly higher in SHRsp than WKY (MWU). (g) Scatterplot SD propagation speed versus mean arterial pressure (MAP). SHRsp and WKY are marked in different colors. SHRsp and WKY appear as two distinct clusters. (h) Scatterplot SD threshold versus MAP. No statistically significant correlation was found. (i) Scatterplot SD propagation speed versus body weight (BW). SHRsp and WKY appear as two distinct clusters. The higher speeds in the lower body weight animals fit well with previous findings in malnourished rodents.^137^ Guedes discussed three main factors as possibly causative: (i) reduction in brain myelin content, (ii) impairment of glial function, and (iii) increase in the cell packing density with reduction of the extracellular space.^138^ and (j) Scatterplot SD threshold versus BW. The statistical relationship in the pooled data is based on the influence of the SHRsp. For the 23 SHRsp alone, the Spearman coefficient was 0.69 (*p* ≤ 0.001), while no significant relationship between SD threshold and BW was found for the 22 WKY [Spearman coefficient: −0.12 (*p* = 0.589)]. The whiskers (error bars) above and below the boxes indicate the 90th and 10th percentiles.

Thresholds to trigger SD were compared only between SHRsp and WKY receiving a regular diet. Experiments from series 1A (Figure 1(b)) and 1B were pooled. We found that the median SD threshold was significantly lower in SHRsp than WKY (Figure 4(a)). In addition, we found that the median propagation speed of SD was significantly faster in SHRsp than WKY (Figure 4(b)). In series 1A, the amplitude of the epidural negative DC shift was not different between SHRsp and WKY (Figure 4(c)). In series 1B, the amplitude of the subdural negative DC shift was not different between SHRsp and WKY (Figure 4(d)). In series 1A, the peak of the LDF-determined hyperemia was not significantly different between SHRsp and WKY (Figure 4(e)). However, in series 1B, we determined the median of 5 ROIs in the window area for each animal and found that the LASCA-determined hyperemia was significantly higher in SHRsp than WKY (Figure 4(f)). Figure 4(g) to (j) examines possible relationships between SD propagation speed and SD threshold, on the one hand, and MAP and BW, on the other hand, for the pooled data of SHRsp and WKY.

### Ex-vivo MRI scan

**Figure 5.**
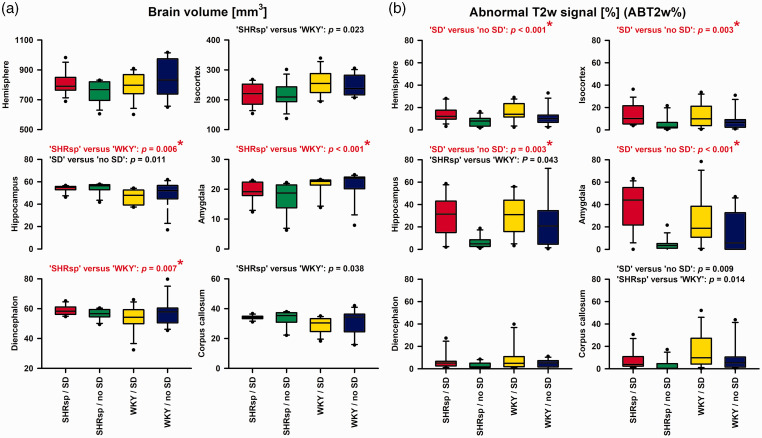
Post-mortem magnetic resonance imaging (MRI) showed on the one hand brain tissue volume differences between stroke-prone spontaneously hypertensive rats (SHRsp) and Wistar-Kyoto rats (WKY) (a), and on the other hand differences in the abnormal T2-weighted (T2w) signal in % of the total volume (ABT2w%) indicating edema between the hemisphere with spreading depolarization (SD) and the contralateral hemisphere without SD (b). For these analyses, we used two-way repeated-measures analysis of variance (ANOVA) on ranks with post-hoc Bonferroni t-tests (SHRsp versus WKY and brain side with versus without SD as factors). Both volumetric data and ABT2w% were compared for the whole hemisphere, isocortex, hippocampus, amygdala, diencephalon and corpus callosum. This means that 6 tests each were performed for the tissue volume variable and the ABT2w% variable, respectively. Therefore, using a strict Bonferroni correction, only *p* values ≤0.05/6 = 0.0083 in (a) and (b) indicate significance. Such tests were marked in red and with a star. X-axes of the four bottom plots also apply to the upper plots. The whiskers (error bars) above and below the boxes indicate the 90th and 10th percentiles. Edema on T2w-MRI of the SD-exposed hemisphere compared with the contralateral side, was most likely vasogenic^74^ and a consequence of blood-brain barrier (BBB) opening to SD.^75–77^ However, in a previous study,^75^ it was found that the SD-induced opening of the BBB to large molecules measured by Evans blue extravasation is mediated by increased endothelial transcytosis, which only starts between 3 and 6 hours, whereas here we found evidence of vasogenic edema as early as 2 hours. In this regard, we cannot exclude the possibility that already the creation of the burr holes in our experiments promoted edema development, although the dura remained closed. However, it could also be that T2w-MRI is more sensitive than Evans blue extravasation. Discrepancies with the time course of BBB opening determined with Evans blue also exist when, for example, Na^+^ fluorescein is used, which indicates a BBB opening in the ischemic penumbra several hours before the BBB opening detected with Evans blue.^139^ With strict Bonferroni correction, the hippocampus and amygdala, in addition to the isocortex, showed a significant ABT2w% signal in the hemisphere where the SD occurred. Both are, in principle, structures that can be reached via gray matter connections from an SD beginning in the isocortex, although the hurdle to reach the hippocampus appears to be higher than the hurdle to reach the amygdala.^140,141^ At older ages, SHRsp typically show local BBB leakage,^60^ but in the present study in comparatively young animals and in a very early time window, we found no evidence that SHRsp have a higher vasogenic edema tendency compared with WKY.

Overall, post-mortem MRIs showed structural changes after the experiments. Thus, lateral ventricles enlarged in comparison to lateral ventricles of naïve rats (Figure 1(c)). In each hemisphere, we analyzed cortex, hippocampus, amygdala, diencephalon, and corpus callosum. For these structures, (i) volumetric differences of the respective structure and (ii) differences in the percentage of voxels with hyperintense (abnormal) T2w signal (ABT2w%) were compared for the between-subjects variable (SHRsp versus WKY) and the within-subjects variable (hemisphere that was electrically stimulated versus contralateral hemisphere) using two-way repeated-measures ANOVA on ranks with post-hoc Bonferroni t-tests. Significant volumetric differences were found for hippocampus, amygdala and diencephalon between SHRsp and WKY (Figure 5(a)). In contrast, significant differences in ABT2w% indicating edema were found for the whole hemisphere, isocortex, hippocampus, and amygdala between the hemisphere that was electrically stimulated and the contralateral hemisphere but not between SHRsp and WKY (Figure 5(b)). The side on which SD occurred showed more edema than the contralateral side.

## SD-induced spreading ischemia

**Figure 6.**
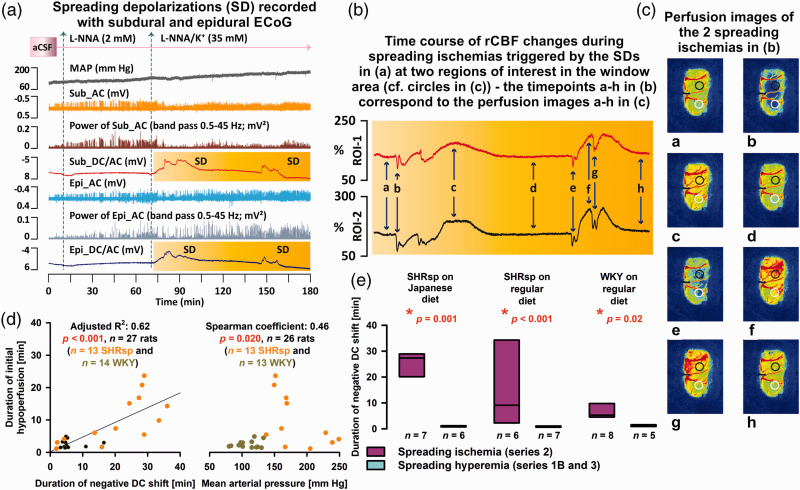
In series 2, brain topical application of artificial cerebrospinal fluid (aCSF) containing the nitric oxide synthase (NOS) inhibitor N^G^-nitro-L-arginine (L-NNA) and increased K^+^ concentration ([K^+^]_aCSF_) induced spreading ischemia. (a) Original recording of an experiment in a stroke-prone spontaneously hypertensive rat (SHRsp) on Japanese diet of series 2. Trace 1 from top to bottom shows the mean arterial pressure (MAP). Traces 2 and 3 demonstrate the depressive effect of the spreading depolarizations (SD) on the spontaneous brain activity as assessed in the higher frequency band [alternating current (AC)-electrocorticography (ECoG), bandpass: 0.5–45 Hz] of the subdural (Sub) recordings. Trace 4 gives the direct current (DC)/AC-ECoG recordings (bandpass: 0–45 Hz) at the subdural electrode within the cranial window. The SDs are observed as negative DC shifts. Traces 5 and 6 demonstrate the depressive effect of the SDs on the spontaneous brain activity as assessed with the recordings of the epidural (Epi) electrode outside the window area. Trace 7 gives the DC/AC-ECoG at the epidural electrode. In traces 4 and 7, the time period is outlined in yellow for which laser speckle contrast analysis (LASCA) imaging-recorded regional cerebral blood flow (rCBF) in two regions of interest (ROI) in the window area is shown in (b). (b) shows rCBF traces typical for SD-induced spreading ischemias with a prolonged initial hypoperfusion followed by hyperperfusion. In principle, the two events each consist of two SDs, each leading to an initial hypoperfusion due to a vasocontrictive response.^5,6^ Time points a-h in (b) correspond to LASCA perfusion maps a-h in (c). The two circles in (c) show the two ROIs for which rCBF is shown in (b). (d) The left panel shows the linear relationship between duration of initial hypoperfusion and duration of negative DC shift during SD-induced spreading ischemia for the pooled experiments from series 2. The right panel shows that higher blood pressure correlated with prolonged SD-induced spreading ischemia when the experiments from series 2 were pooled. (e) The negative DC shift of SD was always significantly longer when SD in series 2 induced spreading ischemia than when SD in series 1B or 3 induced normal spreading hyperemia (Mann-Whitney Rank Sum Tests).

**Figure 7.**
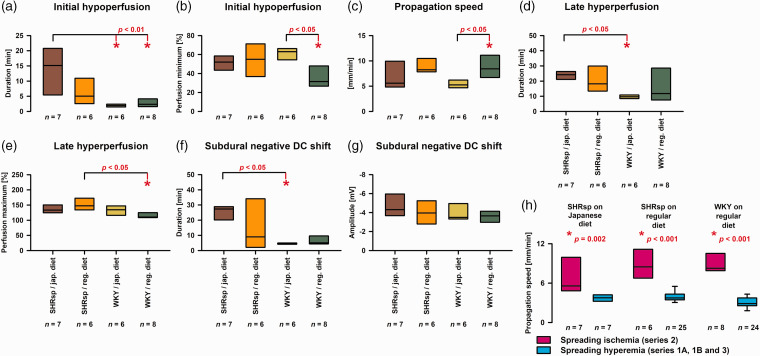
Statistical comparisons of spreading ischemia variables between stroke-prone spontaneously hypertensive rats (SHRsp) on Japanese diet, SHRsp on regular diet, Wistar-Kyoto rats (WKY) on Japanese diet and WKY on regular diet. The analysis of these variables has previously been explained using original traces of a spreading ischemia in a Wistar rat in figure 2 in literature.^64^ (a) For the one-way analysis of variance (ANOVA) with post-hoc Bonferroni t-tests, the duration of the initial hypoperfusion in response to spreading depolarization (SD) was submitted to logarithmic transformation to approach normal distribution [SHRsp on Japanese diet versus (1) WKY on Japanese diet: *p* = 0.003, and versus (2) WKY on regular diet: *p* = 0.006]. (b) The perfusion minima during spreading ischemia showed a significant difference between WKY on Japanese diet and regular diet (one-way ANOVA with Bonferroni t-test: *p* = 0.013). When evaluating perfusion minima, it is important to remember that baseline blood flow in the brain is already reduced by approximately 40% compared with normal blood flow in awake animals because of barbiturate anesthesia.^142,143^ (c) The propagation speed of SD-induced spreading ischemia showed a significant difference between WKY on Japanese diet and regular diet (one-way ANOVA with Bonferroni t-test: *p* = 0.040). (d) The duration of the late hyperperfusion following the initial hypoperfusion during spreading ischemia differed significantly between SHRsp and WKY on Japanese diet [Kruskal-Wallis One Way Analysis of Variance on Ranks (KW-ANOVA) and post-hoc Dunn’s test (phD)]. (e) The perfusion maxima of the late hyperperfusion following the initial hypoperfusion differed significantly between SHRsp and WKY on regular diet (ANOVA with Bonferroni t-test: *p* = 0.020). (f) The duration of the negative subdural DC shift of the spreading ischemia-inducing SD differed significantly between SHRsp and WKY on Japanese diet (KW-ANOVA and phD). (g) The amplitude of the negative subdural DC shift of the spreading ischemia-inducing SD did not differ significantly between the four different groups of animals (one-way ANOVA: *p* = 0.404) and (h) The propagation speed of SD-induced spreading ischemia in series 2 was significantly higher than the propagation speed of SD-induced spreading hyperemia in series 1B or 3 (Mann-Whitney Rank Sum Tests).

Brain topical application of L-NNA and elevated [K^+^]_aCSF_ led to spontaneously recurring SDs (Figure 6(a)) that induced spreading ischemias in all groups (Figure 6(b) and (c)). Supplementary Video 1 shows the LASCA-recorded perfusion changes of the first SD-induced spreading ischemia of Figure 6(a) to (c). In SHRsp with Japanese diet, SHRsp with regular diet, and WKY with regular diet, we found that the negative DC shift was always significantly longer when SD in series 2 induced spreading ischemia than when SD in series 1B or 3 induced normal spreading hyperemia (Figure 6(e)). In the left panel of Figure 6(d), the resulting linear relationship between duration of initial hypoperfusion and duration of negative DC shift is shown for the pooled experiments from series 2. The same linear relationship has been demonstrated for the spreading ischemia continuum previously in other settings, including experimental models and patients with aSAH.^9,69,70^ Interestingly, systemic hypotension alone even in the absence of concomitant NOS inhibition and elevated [K^+^]_aCSF_ led to initial hypoperfusion in response to SD in previous experiments in rats.^71^ However, when considering the mixed population of SHRsp and WKY in series 2, this effect appeared to be reversed, and higher blood pressure correlated with prolonged initial hypoperfusion (right panel in Figure 6(d)). In Figure 7, several variables of the first SD-induced spreading ischemia are compared between the four groups of animals. Figure 7(a) illustrates that the duration of SD-induced initial hypoperfusion was significantly longer in SHRsp on Japanese diet than WKY on either Japanese or regular diet. In addition, the duration of the negative DC shift was significantly longer in SHRsp on Japanese diet than WKY on Japanese diet (Figure 7(f)). The amplitude of the negative DC shift did not differ (Figure 7(g)). Perfusion minima during spreading ischemia or perfusion maxima during hyperemia subsequent to spreading ischemia did not show strong differences (Figure 7(b) and (e)). The propagation speed of SD-induced spreading ischemia showed a significant difference between WKY on Japanese diet and regular diet (Figure 7(c)). More importantly, the propagation speed of SD-induced spreading ischemia in series 2 was significantly higher than the propagation speed of SD-induced spreading hyperemia in series 1A, 1B or 3 in SHRsp with Japanese diet and normal diet and WKY with normal diet (Figure 7(h)).

## SHRsp receiving japanese diet and subjected to physiological aCSF show a normal hemodynamic response to SD

An example for this is given in Supplementary Video 2. The median amplitude of the hyperemic response to SD was 163 (interquartile range (IQR): 159–201) % in this group. The median electrical SD threshold was 2300 (830–2300) mA (n = 6), the median propagation speed 3.7 (3.2–4.0) mm/min (n = 7), and the median amplitude of the subdural negative DC shift 1.8 (1.2–2.5) mV (n = 6).

## Discussion

We found that SHRsp have a significantly lower electrical threshold to trigger SD *in vivo* and show higher SD speed under otherwise physiological conditions. This was investigated under thiopental anesthesia, which is known to enhance the inhibitory action of GABA_A_ receptors. GABA_A_ receptor activation has been shown to limit SD velocity.^72,73^ Although this effect should affect both strains in a similar way, it cannot be excluded that it influenced the result. Edema on T2w-MRI of the SD-exposed hemisphere compared with the contralateral side, was most likely vasogenic^74^ and a consequence of BBB opening to SD,^75–77^ but did not differ between SHRsp and WKY. Consistent with vascular impairment in SHRsp on Japanese diet, SD-induced spreading ischemia lasted significantly longer than in WKY on Japanese or regular diet using a standard model of spreading ischemia. Our results support the hypothesis that in the SHRsp rat model, similar to α_2_NaKA-dysfunctional mice,^50–52^ genetic predisposition to salt-sensitivity is associated with cerebrovascular dysfunction^40,78^ and increased susceptibility to SD.^36–41^ These findings may have implications for a variety of conditions in which SDs occur and hypertension is a vascular risk factor. For example, this applies to delayed cerebral ischemia after aSAH, in which the occurrence of SDs and spreading ischemia has been unequivocally demonstrated in patients^9,10^ and chronic hypertension is considered a risk factor.^79,80^ However, the findings are also interesting for the association between migraine with aura and stroke.^81–87^ The vascular risk factor hypertension, which is significantly more common in migraine patients,^88–91^ has been suggested as one of the possible mediators for this association. However, it may not be mediation in the strict sense, but the basic mechanisms underlying the development of (1) salt-sensitive hypertension, (2) cerebrovascular dysfunction, and (3) SD, respectively, may overlap. In this context, for example, clinical data are interesting, showing that migraine is primarily a disease of premenopausal women but is associated with increased rates of incident hypertension after menopause.^89^

### Salt-sensitivity

High salt intake is attributed to escalating cardio- and cerebrovascular morbidity and mortality. Salt-sensitivity is usually defined as a significant blood pressure response to increased NaCl intake,^42^ whereas KCl intake lowers blood pressure.^92,93^ The Na^+^ sensor(s) that mediate this pressure response have not been fully elucidated. However, tight molecular links are thought to exist between Na^+^ and the dynamic function of vessels, mediating the pressor and depressor effects of high- and low-salt diets.^94^ One link is the RAAS. When salt intake is reduced, RAAS activity enhances the expression of Na^+^ channels and NaKA in the kidney^95,96^ as well as renal Na^+^ reabsorption and vascular and central mechanisms that increase arterial tone and thirst. Nevertheless, the possibility that renal signals drive the blood pressure response to high-salt diet seems unlikely because the circulating RAAS is normally suppressed under high-salt diet.^50^

In contrast, increasing evidence suggests that the Na^+^ sensor and the key to salt-sensitivity are found in the brain. This research essentially began with papers by Takahashi and colleagues in 1988, who proposed that a hypothalamic NaKA inhibitor links brain RAAS activation to the hypertensinogenic effects of salt.^97,98^ Thus, a high-salt diet increases the Na^+^ concentration in the plasma ([Na^+^]_plasma_), and many patients with essential hypertension have a slightly elevated but significant increase in [Na^+^]_plasma_.^99–101^ Moreover, when controlled for age, patients with refractory hypertension had increased tissue Na^+^ content, compared with normotensive controls as assessed using ^23^Na-MRI.^102^ Plasma Na^+^ enters the CSF via the fenestrated capillaries of the circumventricular organs and choroid plexus, and elevated CSF Na^+^ concentration ([Na^+^]_CSF_) then stimulates magnocellular neurosecretary cells of the hypothalamus. Salt uptake from plasma into CSF is physiological and, accordingly, [Na^+^]_CSF_ increased significantly in response to a high-salt diet not only in salt-sensitive but also salt-resistant hypertensive patients.^103^ NaCl uptake from plasma into CSF occurs in a delayed fashion. Thus, intravenous infusion of a small volume of hypertonic saline in healthy dogs resulted in an increase in [Na^+^]_plasma_ from ∼149 to ∼163mM within 15 min, whereas [Na^+^]_CSF_ reached the same elevated value as [Na^+^]_plasma_ only after 90 min.^104^ Importantly, even if [Na^+^]_CSF_ increases in isolation by intracerebroventricular application and [Na^+^]_plasma_ remains unchanged, a short-term pressor response occurs that is associated with enhanced sympathetic activity.^105^ Prolonged administration of NaCl into the CSF led to sustained systemic hypertension.^51^ This pressor response to elevated [Na^+^]_CSF_ is enhanced in various strains of salt-sensitive rodents, including SHRsp, and several lines of experimental evidence suggest that this enhancement involves the central release of endogenous ouabain.^50,52,57^

The specific relevance of the ouabain-binding site on α_2_NaKA in salt-sensitive hypertension is based in particular on studies in genetically modified mice. Thus, in a study of isoflurane-anesthetized α_2_NaKA-deficient knockout mice carrying a null mutation in one ATP1A2 allele, the haploinsufficiency resulted in an increase in baseline blood pressure.^53^ Whereas this effect on baseline blood pressure was not significant when the mice were conscious,^106^ the increased salt-sensitivity was also convincingly demonstrated in conscious α_2_^+/−^ mice.^54,55^ Thus, the pressor response to both an increase in [Na^+^]_CSF_ and intracerebroventricular ouabain was significantly greater in adult α_2_^+/−^ mice than in their WT littermates. And, most importantly, neither increased [Na^+^]_CSF_ nor intracerebroventricular ouabain induced any pressor response in knockout/knockin mice with an ouabain-resistant α_2_NaKA, in contrast to WT animals.^51^ Further indirect support that salt-sensitivity involves the α_2_NaKA comes from the finding that blood from volume-expanded rodents contained increased amounts of a NaKA inhibitor that was not present in animals with electrolytic lesions of the anteroventral third ventricular area (AV3V) of the hypothalamus.^107^ In addition, the development of hypertension in the presence of AV3V lesions does not occur in many animal models of hypertension, and the humoral NaKA inhibitor cannot be detected.^108^ In addition to salt-sensitive hypertension, also adrenocorticotropin-induced hypertension, which is commonly employed as a model of stress-related hypertension, depended on α_2_NaKA.^109^

Overall, elevated [Na^+^]_CSF_ is now thought to cause sustained activation of central angiotensinergic pathways leading to symapthoexcitation and hypertension, which can be prevented by blockade of central AT1Rs.^50^ Sustained activation of these pathways depends on slow neuromodulation in which ENaCs of the ependyma lining the cerebroventricular compartment are activated by binding of aldosterone to central mineralocorticoid receptors. ENaC activation in turn stimulates the production of endogenous ouabain. Endogenous ouabain enhances protein expression of angiotensin converting enzyme, AT1Rs and NADPH oxidase subunits, decreases neuronal NOS and thereby persistently upregulates the central angiotensinergic pressure pathways. Of particular interest in the sustained activation of this neuronal network is that the effect of endogenous ouabain is not mediated by α_3_NaKA on neurons but by α_2_NaKA, which is not present on neurons but only on astrocytes and vascular cells in the adult brain.^50,110,111^

### α_2_NaKA plays a particular role in membrane microdomains

In contrast to α_1_NaKA, α_2_NaKA is not ubiquitously distributed in the plasma membrane but restricted to microdomains neighboring the junctional sarco(endo)plasmic reticulum (jSER).^112,113^ Plasmalemmal Na^+^/Ca^2+^-exchanger (NCX1) and jSER Ca^2+^-ATPase (SERCA) are found in the same microdomains similar to different transient receptor protein canonical proteins as components of receptor- and store-operated cation-selective channels.^112,114^ Altogether, these structures form the plasmerosome as a functional unit in which plasma membrane and jSER are only 12–20 nm apart. As a consequence, Na^+^ and Ca^2+^ diffusion between plasmerosome and cytosol is limited, resulting in [Na^+^] and [Ca^2+^] gradients between plasmerosome and cytosol.^110,113^ Decline in α_2_NaKA activity, e.g., by ouabain, results in increased Ca^2+^ uptake by the jSER because of a decrease in Ca^2+^ efflux via plasmalemmal NCX1. Overall, these changes increase the potential for Ca^2+^ mobilization from the jSER and thus enhance cellular Ca^2+^ signaling.

In addition to these ionic mechanisms at the plasmerosome, α_2_NaKA seems to interact with neighboring membrane proteins and organized cytosolic cascades of signaling proteins to communicate with intracellular organelles.^115^ These signaling pathways are rapidly activated by the interaction of low concentrations of ouabain with α_2_NaKA and independent of changes in cytoplasmic Na^+^ ([Na^+^]_cyt_), K^+^ ([K^+^]_cyt_) and Ca^2+^ ([Ca^2+^]_cyt_) concentrations. They include activation of non-receptor cellular tyrosine protein kinase (cSrc), which leads to tyrosine phosphorylation of a number of cellular proteins and augmented generation of reactive oxygen species (ROS) by mitochondria. The increase in ROS is an essential second messenger for many, though not all, of the downstream events connected with α_2_NaKA. Through these signaling pathways, α_2_NaKA appears to be engaged in multiple regulatory processes affecting cell metabolism, growth, proliferation and migration as well as intercellular communication, i.e., processes typically involving protein phosphorylation/dephosphorylation, gene transcription and translation, as well as Ca^2+^ signaling.^52^ For example, the α_2_NaKA-cSrc kinase pathway was involved in enhanced responses to vasoconstrictors in isolated MCAs of mice carrying a G301R point mutation in one ATP1A2 allele, resulting in α_2_NaKA haploinsufficiency (ATP1A2^+/−G301R^), compared with WT mice.^78^ ATP1A2^+/-G301R^ MCAs showed increased depolarization and stronger sensitization to [Ca^2+^]_cyt_, although the increases of [Ca^2+^]_cyt_ were surprisingly less than in WT. This was associated with increased cSrc activation and increased phosphorylation of the myosin targeting subunit (MYPT1) of myosin light chain phosphatase and abolished by cSrc inhibition.

### α_2_NaKA and SD

In patients, loss-of-function mutations in the ATP1A2 gene cause FHM2, which is clinically characterized by complicated migraine auras.^36,76,116^ Direct clinical evidence from ECoG recordings^117^ and indirect evidence from measurements of rCBF or its surrogates^18,118,119^ suggest that migraine aura is one of the clinical manifestations of SD. Accordingly, ATP1A2^+/R887^ mutant mice showed both reduced electrical threshold for SD and increased SD propagation speed *in vivo*.^37^ It is assumed that this results from enhanced glutamatergic transmission. Thus, α_2_NaKA specifically colocalizes in astrocytes with the glutamate transporters GLAST and GLT1 (EAAT1, EAAT2).^120–123^ Analysis at the ultrastructural level demonstrated that this macromolecular complex occurs preferentially in astrocytic processes around asymmetric glutamatergic synaptic junctions, but not around GABAergic terminals.^120^ Under physiological conditions, glutamate is first transported into the cell together with three Na^+^ ions and one proton following the [Na^+^] gradient, whereupon a K^+^ ion is transported out of the cell in return.^124–126^ Thus, the driving force for glutamate uptake is indirectly provided by the work of α_2_NaKA as it maintains the ion gradients in the corresponding microdomains.

### α_2_NaKA and impaired hemodynamic responses to SD

The normal hemodynamic response to SD in naive cortex is somewhat different in mice than in higher species such as rats, swine, and humans.^31^ The mouse response typically begins with marked initial hypoperfusion, followed by a short peak that barely reaches baseline and renewed, very prolonged rCBF reduction by ∼60%.^127^ Concurrent severe hemoglobin desaturation indicates that O_2_-metabolism becomes at least partially supply limited, and decrease in blood volume implies vasoconstriction as the mechanism.^128^ Thus, in the continuum of higher species between normal spreading hyperemia and spreading ischemia when NVU is impaired, the normal mouse hemodynamic response to SD occupies an intermediate position.^31^ Interestingly, this more vasoconstrictor than vasodilator response to SD was shifted even further toward vasoconstriction in heterozygous α_2_NaKA knockout mice compared with WT mice, an effect that was not observed in either α_1_NaKA or α_3_NaKA heterozygous knockout mice.^40^

With regard to the induction of spreading ischemia with a NOS inhibitor and increased [K^+^]_aCSF_ in rats, it is relevant, that prolonged exposure of tissue to elevated [K^+^]_aCSF_ results in an *ex vivo* detectable decrease in the α_2_/α_3_NaKA fraction and that elevated [K^+^]_aCSF_ can be replaced in the pharmacological protocol for induction of spreading ischemia by an ouabain concentration that inhibits α_2_NaKA but not α_1_NaKA.^56^

In addition to α_2_NaKA-dysfunction, there may be further mechanisms specifically contributing to the impaired recovery from the inverse hemodynamic response to SD in SHRsp on Japanese diet. These include endothelial dysfunction triggered by salt loading because the mitochondrial Na^+^/Ca^2+^ exchanger (NCLX) causes increased Na^+^ import into the mitochondrial matrix, which impedes oxidative phosphorylation, thereby limiting ATP production in endothelial cells.^129,130^ NOS inhibition by L-NNA used to generate spreading ischemia was 91% in a previous study with a similar design.^5^ Mechanisms that further reduce the low residual NOS activity might also contribute to prolonged spreading ischemia. These include impaired regulation of endothelial NOS phosphorylation and activation in SHRsp^61,131^ and salt-loading-triggered endothelial dysfunction as a result of a gut-initiated adaptive immune response mediated by Th17 lymphocytes.^132–134^

### Conclusion

The hypothesis that a mechanistic link exists between (1) salt-sensitive hypertension, (2) SD, and (3) inverse hemodynamic responses was derived mainly from genetically α_2_NaKA-dysfunctional mice. Here, we found these associations in SHRsp, a polygenic rat model of salt-sensitive hypertension and propensity to stroke characterized by augmented ouabain-induced vasoconstriction.^57,59^ Our work is the first step necessary to determine whether these associations apply not only to α_2_NaKA-dysfunctional mice but more generally. A limitation is that we performed deep phenotyping with respect to SD but no specific studies to determine whether the observed phenotype is directly associated with α_2_NaKA dysfunction. To our knowledge, α_2_NaKA functionality has not been sufficiently investigated in SHRsp. However, the ATP1A2 gene was previously found to have restriction fragment length polymorphisms between the genomes of normotensive Sprague-Dawley rats and WKY on the one hand and SHR and SHRsp on the other,^58^ salt-loading was found to decrease myocardial α_2_NaKA in SHRsp but not WKY,^135^ and ouabain-induced vasoconstriction is greatly enhanced in SHRsp compared to WKY, suggesting α_2_NaKA dysfunction.^57,59^ Overall, it needs further investigation whether the phenotype we observed indeed partly or completely results from α_2_NaKA dysfunction or has other causes. In addition, we suggest deep phenotyping related to SD in further genetic models of salt-sensitivity such as Dahl salt-sensitive rats to determine whether the dysfunctional aldosterone-ENaC-endogenous ouabain-AT1R cascade in the brain,^50^ is generally or only partially associated with an increased propensity for SD and inverse hemodynamic responses, as this may offer novel targets for the treatment of a number of important clinical conditions and stroke in particular.^28,31,81^

## Supplementary Material

Supplemental Video 1

Supplemental Video 2

Supplementary material

## List of abbreviations

AC, alternating current; ABT2w, abnormal T2-weighted signal; aCSF, artificial cerebrospinal fluid; Ag/AgCl electrode, silver/silver chloride electrode; α_2_NaKA, α_2_-isoform of Na^+^/K^+^-ATPase; ANOVA, analysis of variance; ARRIVE, Animal Research: Reporting of In Vivo Experiments; aSAH, aneurysmal subarachnoid hemorrhage; AT1R, Angiotensin II receptor type 1; ATP1A2, sodium/potassium-transporting ATPase subunit alpha 2; AV3V, anteroventral third ventricular area of the hypothalamus; BBB, blood-brain barrier; BW, body weight; CaCl_2_, calcium chloride; cSrc, non-receptor cellular tyrosine protein kinase; DC, direct current; EAAT1/EAAT2, excitatory amino acid transporter1/2; ECoG, electrocorticogram; ENaC, epithelial sodium channel; FHM2, familial hemiplegic migraine type 2; FSE, fast spin echo; GABA, gamma-aminobutyric acid; GLAST, glutamate/aspartate transporter (EAAT1); GLT1, glutamate transporter type 1 (EAAT2); jSER, junctional sarco(endo)plasmic reticulum; KCl, potassium chloride; LaGeSo, Landesamt für Gesundheit und Soziales Berlin; LASCA, Laser speckle contrast analysis; LDF, laser-Doppler flowmetry; L-NNA, N^G^-nitro-L-arginine; MAP, mean arterial pressure; MCA, middle cerebral artery; MCAO, middle cerebral artery occlusion; MgCl_2_, magnesium chloride; MHS, malignant hemispheric stroke; MRI, magnetic resonance imaging; MWU, Mann-Whitney U test; MYPT1, myosin phosphatase target subunit 1; N_2_, nitrogen; NaCl, sodium chloride; NADPH, reduced nicotinamide adenine dinucleotide phosphate; NaHCO_3_, sodium bicarbonate; NaKA, Na^+^/K^+^-ATPase; NCLX, mitochondrial Na^+^/Ca^2+^-exchanger; NCX1, plasmalemmal Na^+^/Ca^2+^-exchanger; NOS, nitric oxide synthase; NVU, neurovascular unit; PBS, phosphate-buffered saline; pCO_2_, arterial partial pressure of carbon dioxide; PFA, paraformaldehyde: O_2_, arterial partial pressure of oxygen; p_ti_O_2_, tissue partial pressure of oxygen; RAAS, renin-angiotensin-aldosterone system; rCBF, regional cerebral blood flow; ROI, region of interest; ROS, reactive oxygen species; SD, spreading depolarization; SERCA, sarcoplasmic/endoplasmic reticulum calcium ATPase; SHRsp, stroke-prone spontaneously hypertensive rats; T2w, T2-weighted magnetic resonance imaging; TBI, traumatic brain injury; WKY, Wistar Kyoto rats; WT, wild type.

## References

[1] Santello M Toni N Volterra A. Astrocyte function from information processing to cognition and cognitive impairment. Nat Neurosci 2019; 22: 154–166. 30664773 10.1038/s41593-018-0325-8

[2] Dreier JP Lemale CL Kola V , et al. Spreading depolarization is not an epiphenomenon but the principal mechanism of the cytotoxic edema in various gray matter structures of the brain during stroke. Neuropharmacology 2018; 134: 189–207. 28941738 10.1016/j.neuropharm.2017.09.027

[3] Van Harreveld A. Changes in the diameter of apical dendrites during spreading depression. Am J Physiol 1958; 192: 457–463. 13520934 10.1152/ajplegacy.1958.192.3.457

[4] Kirov SA Fomitcheva IV Sword J. Rapid neuronal ultrastructure disruption and recovery during spreading depolarization-induced cytotoxic edema. Cereb Cortex 2020; 30: 5517–5531. 32483593 10.1093/cercor/bhaa134 PMC7566686

[5] Dreier JP Korner K Ebert N , et al. Nitric oxide scavenging by hemoglobin or nitric oxide synthase inhibition by N-nitro-L-arginine induces cortical spreading ischemia when K^+^ is increased in the subarachnoid space. J Cereb Blood Flow Metab 1998; 18: 978–990. 9740101 10.1097/00004647-199809000-00007

[6] Dreier JP. The role of spreading depression, spreading depolarization and spreading ischemia in neurological disease. Nat Med 2011; 17: 439–447. 21475241 10.1038/nm.2333

[7] Dreier JP Fabricius M Ayata C , et al. Recording, analysis, and interpretation of spreading depolarizations in neurointensive care: review and recommendations of the COSBID research group. J Cereb Blood Flow Metab 2017; 37: 1595–1625. 27317657 10.1177/0271678X16654496 PMC5435289

[8] Leão AAP. Spreading depression of activity in the cerebral cortex. J Neurophysiol 1944; 7: 359–390. 10.1152/jn.1947.10.6.409 20268874

[9] Dreier JP Major S Manning A , et al. Cortical spreading ischaemia is a novel process involved in ischaemic damage in patients with aneurysmal subarachnoid haemorrhage. Brain 2009; 132: 1866–1881. 19420089 10.1093/brain/awp102 PMC2702835

[10] Luckl J Lemale CL Kola V , et al. The negative ultraslow potential, electrophysiological correlate of infarction in the human cortex. Brain 2018; 141: 1734–1752. 29668855 10.1093/brain/awy102 PMC5972557

[11] Bosche B Graf R Ernestus RI , et al. Recurrent spreading depolarizations after subarachnoid hemorrhage decreases oxygen availability in human cerebral cortex. Ann Neurol 2010; 67: 607–617. 20437558 10.1002/ana.21943 PMC2883076

[12] Sugimoto K Nomura S Shirao S , et al. Cilostazol decreases duration of spreading depolarization and spreading ischemia after aneurysmal subarachnoid hemorrhage. Ann Neurol 2018; 84: 873–885. 30341966 10.1002/ana.25361

[13] Dreier JP Winkler MKL Major S , et al. Spreading depolarizations in ischaemia after subarachnoid haemorrhage, a diagnostic phase III study. Brain 2022; 145: 1264–1284. 35411920 10.1093/brain/awab457

[14] Hinzman JM Andaluz N Shutter LA , et al. Inverse neurovascular coupling to cortical spreading depolarizations in severe brain trauma. Brain 2014; 137: 2960–2972. 25154387 10.1093/brain/awu241

[15] Woitzik J Hecht N Pinczolits A , et al. Propagation of cortical spreading depolarization in the human cortex after malignant stroke. Neurology 2013; 80: 1095–1102. 23446683 10.1212/WNL.0b013e3182886932

[16] Van Harreveld A Ochs S. Electrical and vascular concomitants of spreading depression. Am J Physiol 1957; 189: 159–166. 13424710 10.1152/ajplegacy.1957.189.1.159

[17] Lauritzen M. Pathophysiology of the migraine aura. The spreading depression theory. Brain 1994; 117: 199–210. 7908596 10.1093/brain/117.1.199

[18] Hadjikhani N Sanchez Del Rio M Wu O , et al. Mechanisms of migraine aura revealed by functional MRI in human visual cortex. Proc Natl Acad Sci U S A 2001; 98: 4687–4692. 11287655 10.1073/pnas.071582498 PMC31895

[19] Leão AAP. Further observations on the spreading depression of activity in the cerebral cortex. J Neurophysiol 1947; 10: 409–414. 20268874 10.1152/jn.1947.10.6.409

[20] Dreier JP Major S Foreman B , et al. Terminal spreading depolarization and electrical silence in death of human cerebral cortex. Ann Neurol 2018; 83: 295–310. 29331091 10.1002/ana.25147 PMC5901399

[21] Shin HK Dunn AK Jones PB , et al. Vasoconstrictive neurovascular coupling during focal ischemic depolarizations. J Cereb Blood Flow Metab 2006; 26: 1018–1030. 16340958 10.1038/sj.jcbfm.9600252

[22] Strong AJ Anderson PJ Watts HR , et al. Peri-infarct depolarizations lead to loss of perfusion in ischaemic gyrencephalic cerebral cortex. Brain 2007; 130: 995–1008. 17438018 10.1093/brain/awl392

[23] Bere Z Obrenovitch TP Kozak G , et al. Imaging reveals the focal area of spreading depolarizations and a variety of hemodynamic responses in a rat microembolic stroke model. J Cereb Blood Flow Metab 2014 2014; 34: 1695–1705. 10.1038/jcbfm.2014.136 PMC4269732 25074743

[24] Zhao HT Tuohy MC Chow D , et al. Neurovascular dynamics of repeated cortical spreading depolarizations after acute brain injury. Cell Rep 2021; 37: 109794. 34610299 10.1016/j.celrep.2021.109794 PMC8590206

[25] Takeda Y Zhao L Jacewicz M , et al. Metabolic and perfusion responses to recurrent peri-infarct depolarization during focal ischemia in the spontaneously hypertensive rat: dominant contribution of sporadic CBF decrements to infarct expansion. J Cereb Blood Flow Metab 2011; 31: 1863–1873. 21522165 10.1038/jcbfm.2011.62 PMC3185883

[26] Nedergaard M Hansen AJ. Spreading depression is not associated with neuronal injury in the normal brain. Brain Res 1988; 449: 395–398. 3395856 10.1016/0006-8993(88)91062-1

[27] Dreier JP Ebert N Priller J , et al. Products of hemolysis in the subarachnoid space inducing spreading ischemia in the cortex and focal necrosis in rats: a model for delayed ischemic neurological deficits after subarachnoid hemorrhage? J Neurosurg 2000; 93: 658–666. 11014545 10.3171/jns.2000.93.4.0658

[28] Hartings JA Shuttleworth CW Kirov SA , et al. The continuum of spreading depolarizations in acute cortical lesion development: Examining leao's legacy. J Cereb Blood Flow Metab 2017; 37: 1571–1594. 27328690 10.1177/0271678X16654495 PMC5435288

[29] Dijkhuizen RM Beekwilder JP van der Worp HB , et al. Correlation between tissue depolarizations and damage in focal ischemic rat brain. Brain Res 1999; 840: 194–205. 10517971 10.1016/s0006-8993(99)01769-2

[30] Zhao L Nowak TS Jr. Preconditioning cortical lesions reduce the incidence of peri-infarct depolarizations during focal ischemia in the spontaneously hypertensive rat: interaction with prior anesthesia and the impact of hyperglycemia. J Cereb Blood Flow Metab 2015; 35: 1181–1190. 25757750 10.1038/jcbfm.2015.37 PMC4640273

[31] Dreier JP Reiffurth C. The stroke-migraine depolarization continuum. Neuron 2015; 86: 902–922. Review. 25996134 10.1016/j.neuron.2015.04.004

[32] Somjen GG. Mechanisms of spreading depression and hypoxic spreading depression-like depolarization. Physiol Rev 2001; 81: 1065–1096. 11427692 10.1152/physrev.2001.81.3.1065

[33] Chuquet J Hollender L Nimchinsky EA. High-resolution in vivo imaging of the neurovascular unit during spreading depression. J Neurosci 2007; 27: 4036–4044. 17428981 10.1523/JNEUROSCI.0721-07.2007 PMC6672520

[34] Peters O Schipke CG Hashimoto Y , et al. Different mechanisms promote astrocyte Ca2+ waves and spreading depression in the mouse neocortex. J Neurosci 2003; 23: 9888–9896. 14586018 10.1523/JNEUROSCI.23-30-09888.2003 PMC6740882

[35] Largo C Cuevas P Somjen GG , et al. The effect of depressing glial function in rat brain in situ on ion homeostasis, synaptic transmission, and neuron survival. J Neurosci 1996; 16: 1219–1229. 8558250 10.1523/JNEUROSCI.16-03-01219.1996 PMC6578797

[36] De Fusco M Marconi R Silvestri L , et al. Haploinsufficiency of ATP1A2 encoding the Na^+^/K^+^ pump alpha2 subunit associated with familial hemiplegic migraine type 2. Nat Genet 2003; 33: 192–196. 12539047 10.1038/ng1081

[37] Leo L Gherardini L Barone V , et al. Increased susceptibility to cortical spreading depression in the mouse model of familial hemiplegic migraine type 2. PLoS Genet 2011; 7: e1002129. 21731499 10.1371/journal.pgen.1002129 PMC3121757

[38] Parker PD Suryavanshi P Melone M , et al. Non-canonical glutamate signaling in a genetic model of migraine with aura. Neuron 2021; 109: 611–628 e618. 10.1016/j.neuron.2020.11.018 PMC7889497 33321071

[39] Unekawa M Ikeda K Tomita Y , et al. Enhanced susceptibility to cortical spreading depression in two types of Na(+),K(+)-ATPase alpha2 subunit-deficient mice as a model of familial hemiplegic migraine 2. Cephalalgia 2018; 38: 1515–1524. 29041816 10.1177/0333102417738249

[40] Reiffurth C Alam M Zahedi-Khorasani M , et al. K(+)-ATPase alpha isoform deficiency results in distinct spreading depolarization phenotypes. J Cereb Blood Flow Metab 2020; 40: 622–638. 30819023 10.1177/0271678X19833757 PMC7025397

[41] Smith SE Chen X Brier LM , et al. Astrocyte deletion of alpha2-Na/K ATPase triggers episodic motor paralysis in mice via a metabolic pathway. Nat Commun 2020; 11: 6164. 33268780 10.1038/s41467-020-19915-2 PMC7710756

[42] Cuevas S Asico LD Jose PA , et al. Renal hydrogen peroxide production prevents salt-sensitive hypertension. J Am Heart Assoc 2020; 9: e013818. 31902320 10.1161/JAHA.119.013818 PMC6988155

[43] Felder RA White MJ Williams SM , et al. Diagnostic tools for hypertension and salt sensitivity testing. Curr Opin Nephrol Hypertens 2013; 22: 65–76. 23197156 10.1097/MNH.0b013e32835b3693 PMC3724405

[44] Okamoto K Aoki K. Development of a strain of spontaneously hypertensive rats. Jpn Circ J 1963; 27: 282–293. 13939773 10.1253/jcj.27.282

[45] Yamori Y Nagaoka A Okamoto K. Importance of genetic factors in hypertensive cerebrovascular lesions; an evidence obtained by successive selective breeding of stroke-prone and -resistant SHR. Jpn Circ J 1974; 38: 1095–1100. 4436938 10.1253/jcj.38.1095

[46] Maier B Kubis N. Hypertension and its impact on stroke recovery: from a vascular to a parenchymal overview. Neural Plast 2019; 2019: 6843895. 31737062 10.1155/2019/6843895 PMC6815533

[47] Griffin KA Churchill PC Picken M , et al. Differential salt-sensitivity in the pathogenesis of renal damage in SHR and stroke prone SHR. Am J Hypertens 2001; 14: 311–320. 11336176 10.1016/s0895-7061(00)01282-6

[48] Nagaoka A Iwatsuka H Suzuoki Z , et al. Genetic predisposition to stroke in spontaneously hypertensive rats. Am J Physiol 1976; 230: 1354–1359. 1275077 10.1152/ajplegacy.1976.230.5.1354

[49] Rubattu S Stanzione R Volpe M. Mitochondrial dysfunction contributes to hypertensive target organ damage: Lessons from an animal model of human disease. Oxid Med Cell Longev 2016; 2016: 1067801–1067809. 27594970 10.1155/2016/1067801 PMC4993945

[50] Leenen FHH Wang HW Hamlyn JM. Sodium pumps, ouabain and aldosterone in the brain: a neuromodulatory pathway underlying salt-sensitive hypertension and heart failure. Cell Calcium 2020; 86: 102151. 31954234 10.1016/j.ceca.2019.102151

[51] Van Huysse JW Dostanic I Lingrel JB , et al. Hypertension from chronic Central sodium chloride in mice is mediated by the ouabain-binding site on the Na,K-ATPase alpha(2)-isoform. Am J Physiol Heart Circ Physiol 2011; 301: H2147–2153. 21856907 10.1152/ajpheart.01216.2010 PMC3213960

[52] Blaustein MP Hamlyn JM. Ouabain, endogenous ouabain and ouabain-like factors: the Na(+) pump/ouabain receptor, its linkage to NCX, and its myriad functions. Cell Calcium 2020; 86: 102159. 31986323 10.1016/j.ceca.2020.102159

[53] Zhang J Lee MY Cavalli M , et al. Sodium pump alpha2 subunits control myogenic tone and blood pressure in mice. J Physiol 2005; 569: 243–256. 16166162 10.1113/jphysiol.2005.091801 PMC1464198

[54] Hou X Theriault SF Dostanic-Larson I , et al. Enhanced pressor response to increased CSF sodium concentration and to central ANG I in heterozygous alpha2 Na+-K+-ATPase knockout mice. Am J Physiol Regul Integr Comp Physiol 2009; 296: R1427–1438. 19244589 10.1152/ajpregu.00809.2007 PMC2689841

[55] Van Huysse JW. Endogenous brain Na pumps, brain ouabain-like substance and the alpha2 isoform in salt-dependent hypertension. Pathophysiology 2007; 14: 213–220. 17980562 10.1016/j.pathophys.2007.09.010

[56] Major S Petzold GC Reiffurth C , et al. A role of the sodium pump in spreading ischemia in rats. J Cereb Blood Flow Metab 2017; 37: 1687–1705. 26994042 10.1177/0271678X16639059 PMC5435275

[57] Imaizumi T Takeshita A Ashihara T , et al. Augmented vascular responses to ouabain in SHRSP during salt loading. Clin Exp Hypertens A 1987; 9: 753–771. 2441910 10.3109/10641968709161448

[58] Nojima H Yagawa Y Kawakami K. The Na,K-ATPase alpha 2 subunit gene displays restriction fragment length polymorphisms between the genomes of normotensive and hypertensive rats. J Hypertens 1989; 7: 937–940. 2576429 10.1097/00004872-198912000-00002

[59] Bruner CA Myers JH Sing CF , et al. Genetic basis for altered vascular responses to ouabain and potassium-free solution in hypertension. Am J Physiol 1986; 251: H1276–1282. 3789180 10.1152/ajpheart.1986.251.6.H1276

[60] Hainsworth AH Markus HS. Do in vivo experimental models reflect human cerebral small vessel disease? A systematic review. J Cereb Blood Flow Metab 2008; 28: 1877–1891. 18698331 10.1038/jcbfm.2008.91

[61] Daneshtalab N Smeda JS. Alterations in the modulation of cerebrovascular tone and blood flow by nitric oxide synthases in SHRsp with stroke. Cardiovasc Res 2010; 86: 160–168. 20008826 10.1093/cvr/cvp395

[62] Enea I De Paolis P Porcellini A , et al. Defective suppression of the aldosterone biosynthesis during stroke permissive diet in the stroke-prone phenotype of the spontaneously hypertensive rat. Basic Res Cardiol 2000; 95: 84–92. 10826499 10.1007/s003950050168

[63] Yamori Y Horie R Tanase H , et al. Possible role of nutritional factors in the incidence of cerebral lesions in stroke-prone spontaneously hypertensive rats. Hypertension 1984; 6: 49–53. 6693147 10.1161/01.hyp.6.1.49

[64] Dreier JP Petzold G Tille K , et al. Ischaemia triggered by spreading neuronal activation is inhibited by vasodilators in rats. J Physiol 2001; 531: 515–526. 11230523 10.1111/j.1469-7793.2001.0515i.x PMC2278483

[65] Rubattu S Hubner N Ganten U , et al. Reciprocal congenic lines for a major stroke QTL on rat chromosome 1. Physiol Genomics 2006; 27: 108–113. 16835352 10.1152/physiolgenomics.00086.2006

[66] Schoknecht K Kikhia M Lemale CL , et al. The role of spreading depolarizations and electrographic seizures in early injury progression of the rat photothrombosis stroke model. J Cereb Blood Flow Metab 2021; 41: 413–430. 32241203 10.1177/0271678X20915801 PMC7812510

[67] Oliveira-Ferreira AI Milakara D Alam M , COSBID study group et al. Experimental and preliminary clinical evidence of an ischemic zone with prolonged negative DC shifts surrounded by a normally perfused tissue belt with persistent electrocorticographic depression. J Cereb Blood Flow Metab 2010; 30: 1504–1519. 20332797 10.1038/jcbfm.2010.40 PMC2949249

[68] Kang EJ Major S Jorks D , et al. Blood-brain barrier opening to large molecules does not imply blood-brain barrier opening to small ions. Neurobiol Dis 2013; 52: 204–218. 23291193 10.1016/j.nbd.2012.12.007

[69] Dreier JP Windmuller O Petzold G , et al. Ischemia caused by inverse coupling between neuronal activation and cerebral blood flow in rats. In: Tomita M Kanno I Hamel E (eds) Brain activation and CBF control. Amsterdam: Elsevier, 2002, pp.487–492.

[70] Bere Z Obrenovitch TP Bari F , et al. Ischemia-induced depolarizations and associated hemodynamic responses in incomplete global forebrain ischemia in rats. Neuroscience 2014; 260: 217–226. 24365459 10.1016/j.neuroscience.2013.12.032

[71] Sukhotinsky I Dilekoz E Moskowitz MA , et al. Hypoxia and hypotension transform the blood flow response to cortical spreading depression from hyperemia into hypoperfusion in the rat. J Cereb Blood Flow Metab 2008; 28: 1369–1376. 18446167 10.1038/jcbfm.2008.35

[72] Aiba I Carlson AP Sheline CT , et al. Synaptic release and extracellular actions of Zn2^+^ limit propagation of spreading depression and related events in vitro and in vivo. J Neurophysiol 2012; 107: 1032–1041. 22131381 10.1152/jn.00453.2011 PMC3289481

[73] Guedes RC Barreto JM. Effect of anesthesia on the propagation of cortical spreading depression in rats. Braz J Med Biol Res 1992; 25: 393–397. 1342216

[74] Bar-Klein G Lublinsky S Kamintsky L , et al. Imaging blood-brain barrier dysfunction as a biomarker for epileptogenesis. Brain 2017; 140: 1692–1705. 28444141 10.1093/brain/awx073

[75] Sadeghian H Lacoste B Qin T , et al. Spreading depolarizations trigger caveolin-1-dependent endothelial transcytosis. Ann Neurol 2018; 84: 409–423. 30014540 10.1002/ana.25298 PMC6153037

[76] Dreier JP Jurkat-Rott K Petzold GC , et al. Opening of the blood-brain barrier preceding cortical edema in a severe attack of FHM type II. Neurology 2005; 64: 2145–2147. 15985592 10.1212/01.WNL.0000176298.63840.99

[77] Gursoy-Ozdemir Y Qiu J Matsuoka N , et al. Cortical spreading depression activates and upregulates MMP-9. J Clin Invest 2004; 113: 1447–1455. 15146242 10.1172/JCI21227 PMC406541

[78] Staehr C Hangaard L Bouzinova EV , et al. Smooth muscle Ca(2+) sensitization causes hypercontractility of Middle cerebral arteries in mice bearing the familial hemiplegic migraine type 2 associated mutation. J Cereb Blood Flow Metab 2019; 39: 1570–1587. 29513112 10.1177/0271678X18761712 PMC6681533

[79] de Rooij NK Rinkel GJ Dankbaar JW , et al. Delayed cerebral ischemia after subarachnoid hemorrhage: a systematic review of clinical, laboratory, and radiological predictors. Stroke 2013; 44: 43–54. 23250997 10.1161/STROKEAHA.112.674291

[80] Ohman J Servo A Heiskanen O. Risks factors for cerebral infarction in good-grade patients after aneurysmal subarachnoid hemorrhage and surgery: a prospective study. J Neurosurg 1991; 74: 14–20. 1984496 10.3171/jns.1991.74.1.0014

[81] Lemale CL Luckl J Horst V , et al. Migraine aura, transient ischemic attacks, stroke, and dying of the brain share the same key pathophysiological process in neurons driven by Gibbs-Donnan forces, namely spreading depolarization. Front Cell Neurosci 2022; 16837650. 10.3389/fncel.2022.837650 PMC8884062 35237133

[82] Oie LR Kurth T Gulati S , et al. Migraine and risk of stroke. J Neurol Neurosurg Psychiatry 2020; 91: 593–604. 32217787 10.1136/jnnp-2018-318254 PMC7279194

[83] Etminan M Takkouche B Isorna FC , et al. Risk of ischaemic stroke in people with migraine: systematic review and meta-analysis of observational studies. Bmj 2005; 330: 63. 15596418 10.1136/bmj.38302.504063.8F PMC543862

[84] Schurks M Rist PM Bigal ME , et al. Migraine and cardiovascular disease: systematic review and Meta-analysis. BMJ 2009; 339: b3914. 19861375 10.1136/bmj.b3914 PMC2768778

[85] Spector JT Kahn SR Jones MR , et al. Migraine headache and ischemic stroke risk: an updated meta-analysis. Am J Med 2010; 123: 612–624. 20493462 10.1016/j.amjmed.2009.12.021 PMC2900472

[86] Hu X Zhou Y Zhao H , et al. Migraine and the risk of stroke: an updated meta-analysis of prospective cohort studies. Neurol Sci 2017; 38: 33–40. 27785579 10.1007/s10072-016-2746-z

[87] Mahmoud AN Mentias A Elgendy AY , et al. Migraine and the risk of cardiovascular and cerebrovascular events: a meta-analysis of 16 cohort studies including 1 152 407 subjects. BMJ Open 2018; 8: e020498. 10.1136/bmjopen-2017-020498 PMC5875642 29593023

[88] Gardener H Monteith T Rundek T , et al. Hypertension and migraine in the Northern Manhattan study. Ethn Dis 2016; 26: 323–330. 27440971 10.18865/ed.26.3.323 PMC4948798

[89] MacDonald CJ El Fatouhi D Madika AL , et al. Association of migraine With incident hypertension after menopause: a longitudinal cohort study. Neurology 2021; 97: e34–e41. 33883242 10.1212/WNL.0000000000011986

[90] Scher AI Terwindt GM Picavet HS , et al. Cardiovascular risk factors and migraine: the GEM population-based study. Neurology 2005; 64: 614–620. 15728281 10.1212/01.WNL.0000151857.43225.49

[91] Tietjen GE Maly EF. Migraine and ischemic stroke in women. A narrative review. Headache 2020; 60: 843–863. 32246455 10.1111/head.13796

[92] Neal B Wu Y Feng X , et al. Effect of salt substitution on cardiovascular events and death. N Engl J Med 2021; 385: 1067–1077. 34459569 10.1056/NEJMoa2105675

[93] Dietz R Schomig A Dart AM , et al. Modulation of sympathetic vasoconstriction by potassium. J Cardiovasc Pharmacol 1984; 6 Suppl 1: S230–235. 6204147 10.1097/00005344-198400061-00036

[94] Hamlyn JM Blaustein MP. Salt sensitivity, endogenous ouabain and hypertension. Curr Opin Nephrol Hypertens 2013; 22: 51–58. 23207724 10.1097/MNH.0b013e32835b36ec PMC3712615

[95] Bonvalet JP. Regulation of sodium transport by steroid hormones. Kidney Int Suppl 1998; 65: S49–56. 9551432

[96] Horisberger JD Rossier BC. Aldosterone regulation of gene transcription leading to control of ion transport. Hypertension 1992; 19: 221–227. 1372288 10.1161/01.hyp.19.3.221

[97] Takahashi H Matsusawa M Suga K , et al. Hypothalamic digitalis-like substance is released with sodium-loading in rats. Am J Hypertens 1988; 1: 146–151. 3401353 10.1093/ajh/1.2.146

[98] Takahashi H Matsusawa M Ikegaki I , et al. Brain renin-angiotensin system and the hypothalamic, digitalis-like Na+, K+-ATPase inhibitor in rats. Clin Exp Hypertens A 1988; 10: 1285–1287. 2852080 10.1080/07300077.1988.11878920

[99] He FJ Markandu ND Sagnella GA , et al. Plasma sodium: ignored and underestimated. Hypertension 2005; 45: 98–102. 15557392 10.1161/01.HYP.0000149431.79450.a2

[100] Suckling RJ He FJ Markandu ND , et al. Dietary salt influences postprandial plasma sodium concentration and systolic blood pressure. Kidney Int 2012; 81: 407–411. 22048126 10.1038/ki.2011.369

[101] de Wardener HE He FJ MacGregor GA. Plasma sodium and hypertension. Kidney Int 2004; 66: 2454–2466. 15569339 10.1111/j.1523-1755.2004.66018.x

[102] Kopp C Linz P Dahlmann A , et al. 23Na magnetic resonance imaging-determined tissue sodium in healthy subjects and hypertensive patients. Hypertension 2013; 61: 635–640. 23339169 10.1161/HYPERTENSIONAHA.111.00566

[103] Kawano Y Yoshida K Kawamura M , et al. Sodium and noradrenaline in cerebrospinal fluid and blood in salt-sensitive and non-salt-sensitive essential hypertension. Clin Exp Pharmacol Physiol 1992; 19: 235–241. 1516270 10.1111/j.1440-1681.1992.tb00444.x

[104] Ito N Suzuki K Koie H , et al. The effect of 7.2% hypertonic saline solution on the duration of sodium gradient between the cerebrospinal fluid and the venous circulation in the dog. J Vet Med Sci 2006; 68: 183–185. 16520544 10.1292/jvms.68.183

[105] Shah J Jandhyala BS. Studies on the role(s) of cerebrospinal fluid osmolality and chloride ion in the centrally mediated pressor responses of sodium chloride. Clin Exp Hypertens A 1991; 13: 297–312. 1829660 10.3109/10641969109042064

[106] James PF Grupp IL Grupp G , et al. Identification of a specific role for the Na,K-ATPase alpha 2 isoform as a regulator of calcium in the heart. Mol Cell 1999; 3: 555–563. 10360172 10.1016/s1097-2765(00)80349-4

[107] Bealer SL Haywood JR Gruber KA , et al. Preoptic-hypothalamic periventricular lesions reduce natriuresis to volume expansion. Am J Physiol 1983; 244: R51–57. 6401408 10.1152/ajpregu.1983.244.1.R51

[108] Brody MJ. Central nervous system and mechanisms of hypertension. Clin Physiol Biochem 1988; 6: 230–239. 3060299

[109] Lorenz JN Loreaux EL Dostanic-Larson I , et al. ACTH-induced hypertension is dependent on the ouabain-binding site of the alpha2-Na+-K+-ATPase subunit. Am J Physiol Heart Circ Physiol 2008; 295: H273–280. 18487447 10.1152/ajpheart.00183.2008 PMC2494766

[110] Blaustein MP Hamlyn JM. Signaling mechanisms that link salt retention to hypertension: endogenous ouabain, the Na(+) pump, the Na(+)/Ca(2+) exchanger and TRPC proteins. Biochim Biophys Acta 2010; 1802: 1219–1229. 20211726 10.1016/j.bbadis.2010.02.011 PMC2909369

[111] Blanco G. Na,K-ATPase subunit heterogeneity as a mechanism for tissue-specific ion regulation. Semin Nephrol 2005; 25: 292–303. 16139684 10.1016/j.semnephrol.2005.03.004

[112] Juhaszova M Blaustein MP. Distinct distribution of different Na+ pump alpha subunit isoforms in plasmalemma. Physiological implications. Ann N Y Acad Sci 1997; 834: 524–536. 9405854 10.1111/j.1749-6632.1997.tb52310.x

[113] Blaustein MP Juhaszova M Golovina VA , et al. Na/Ca exchanger and PMCA localization in neurons and astrocytes: functional implications. Ann N Y Acad Sci 2002; 976: 356–366. 12502582 10.1111/j.1749-6632.2002.tb04762.x

[114] Pulina MV Zulian A Berra-Romani R , et al. Upregulation of Na+ and Ca2+ transporters in arterial smooth muscle from ouabain-induced hypertensive rats. Am J Physiol Heart Circ Physiol 2010; 298: H263–274. 19897708 10.1152/ajpheart.00784.2009 PMC2806143

[115] Xie Z Askari A. Na(+)/K(+)-ATPase as a signal transducer. Eur J Biochem 2002; 269: 2434–2439. 12027880 10.1046/j.1432-1033.2002.02910.x

[116] Jurkat-Rott K Freilinger T Dreier JP , et al. Variability of familial hemiplegic migraine with novel A1A2 Na+/K+-ATPase variants. Neurology 2004; 62: 1857–1861. 15159495 10.1212/01.wnl.0000127310.11526.fd

[117] Major S Huo S Lemale CL , et al. Direct electrophysiological evidence that spreading depolarization-induced spreading depression is the pathophysiological correlate of the migraine aura and a review of the spreading depolarization continuum of acute neuronal mass injury. Geroscience 2020; 42: 57–80. 31820363 10.1007/s11357-019-00142-7 PMC7031471

[118] Olesen J Larsen B Lauritzen M. Focal hyperemia followed by spreading oligemia and impaired activation of rCBF in classic migraine. Ann Neurol 1981; 9: 344–352. 6784664 10.1002/ana.410090406

[119] Lauritzen M, S Olsen T Lassen NA , et al. Changes in regional cerebral blood flow during the course of classic migraine attacks. Ann Neurol 1983; 13: 633–641. 6881926 10.1002/ana.410130609

[120] Cholet N Pellerin L Magistretti PJ , et al. Similar perisynaptic glial localization for the Na+,K+-ATPase alpha 2 subunit and the glutamate transporters GLAST and GLT-1 in the rat somatosensory cortex. Cereb Cortex 2002; 12: 515–525. 11950769 10.1093/cercor/12.5.515

[121] Rose EM Koo JC Antflick JE , et al. Glutamate transporter coupling to Na,K-ATPase. J Neurosci 2009; 29: 8143–8155. 19553454 10.1523/JNEUROSCI.1081-09.2009 PMC6666056

[122] Melone M Ciriachi C Pietrobon D , et al. Heterogeneity of astrocytic and neuronal GLT-1 at cortical excitatory synapses, as revealed by its colocalization With Na+/K+-ATPase alpha isoforms. Cereb Cortex 2019; 29: 3331–3350. 30260367 10.1093/cercor/bhy203

[123] Capuani C Melone M Tottene A , et al. Defective glutamate and K+ clearance by cortical astrocytes in familial hemiplegic migraine type 2. EMBO Mol Med 2016; 8: 967–986. 27354390 10.15252/emmm.201505944 PMC4967947

[124] Kanner BI. Structure and function of sodium-coupled GABA and glutamate transporters. J Membr Biol 2006; 213: 89–100. 17417704 10.1007/s00232-006-0877-5

[125] Wadiche JI Amara SG Kavanaugh MP. Ion fluxes associated with excitatory amino acid transport. Neuron 1995; 15: 721–728. 7546750 10.1016/0896-6273(95)90159-0

[126] Bergles DE Jahr CE. Synaptic activation of glutamate transporters in hippocampal astrocytes. Neuron 1997; 19: 1297–1308. 9427252 10.1016/s0896-6273(00)80420-1

[127] Ayata C Shin HK Salomone S , et al. Pronounced hypoperfusion during spreading depression in mouse cortex. J Cereb Blood Flow Metab 2004; 24: 1172–1182. 15529018 10.1097/01.WCB.0000137057.92786.F3

[128] Yuzawa I Sakadzic S Srinivasan VJ , et al. Cortical spreading depression impairs oxygen delivery and metabolism in mice. J Cereb Blood Flow Metab 2012; 32: 376–386. 22008729 10.1038/jcbfm.2011.148 PMC3272607

[129] Hernansanz-Agustin P Choya-Foces C Carregal-Romero S , et al. Na(+) controls hypoxic signalling by the mitochondrial respiratory chain. Nature 2020; 586: 287–291. 32728214 10.1038/s41586-020-2551-y PMC7992277

[130] Geisberger S Bartolomaeus H Neubert P , et al. Salt transiently inhibits mitochondrial energetics in mononuclear phagocytes. Circulation 2021; 144: 144–158. 33906377 10.1161/CIRCULATIONAHA.120.052788 PMC8270232

[131] Yoshitomi H Xu Q Gao M , et al. Phosphorylated endothelial NOS Ser1177 via the PI3K/akt pathway is depressed in the brain of stroke-prone spontaneously hypertensive rat. J Stroke Cerebrovasc Dis 2011; 20: 406–412. 20813549 10.1016/j.jstrokecerebrovasdis.2010.01.014

[132] Kleinewietfeld M Manzel A Titze J , et al. Sodium chloride drives autoimmune disease by the induction of pathogenic TH17 cells. Nature 2013; 496: 518–522. 23467095 10.1038/nature11868 PMC3746493

[133] Faraco G Brea D Garcia-Bonilla L , et al. Dietary salt promotes neurovascular and cognitive dysfunction through a gut-initiated TH17 response. Nat Neurosci 2018; 21: 240–249. 29335605 10.1038/s41593-017-0059-z PMC6207376

[134] Faraco G Hochrainer K Segarra SG , et al. Dietary salt promotes cognitive impairment through tau phosphorylation. Nature 2019; 574: 686–690. 31645758 10.1038/s41586-019-1688-z PMC7380655

[135] Quintas LE Noel F Wibo M. Na+/K+-ATPase alpha isoforms expression in stroke-prone spontaneously hypertensive rat heart ventricles: effect of salt loading and lacidipine treatment. Eur J Pharmacol 2007; 565: 151–157. 17451677 10.1016/j.ejphar.2007.03.017

[136] Hartings JA Strong AJ Fabricius M , et al. Spreading depolarizations and late secondary insults after traumatic brain injury. J Neurotrauma 2009; 26: 1857–1866. 19508156 10.1089/neu.2009.0961 PMC2865988

[137] Rocha-de-Melo AP Guedes RC. Spreading depression is facilitated in adult rats previously submitted to short episodes of malnutrition during the lactation period. Braz J Med Biol Res 1997; 30: 663–669. 9283636 10.1590/s0100-879x1997000500015

[138] Guedes RCA. Cortical spreading depression: a model for studying brain consequences of malnutrition. In: Preedy VR Watson RR Martin CR (eds) Handbook of behavior, food and nutrition. London: Springer, 2011, pp.2343–2355.

[139] Schoknecht K Prager O Vazana U , et al. Monitoring stroke progression: in vivo imaging of cortical perfusion, blood-brain barrier permeability and cellular damage in the rat photothrombosis model. J Cereb Blood Flow Metab 2014; 34: 1791–1801. 25160672 10.1038/jcbfm.2014.147 PMC4269756

[140] Vinogradova LV Koroleva VI Bures J. Re-entry waves of leao's spreading depression between neocortex and caudate nucleus. Brain Res 1991; 538: 161–164. 2018928 10.1016/0006-8993(91)90392-9

[141] Cain SM Bohnet B LeDue J , et al. In vivo imaging reveals that pregabalin inhibits cortical spreading depression and propagation to subcortical brain structures. Proc Natl Acad Sci U S A 2017; 114: 2401–2406. 28223480 10.1073/pnas.1614447114 PMC5338525

[142] Linde R Schmalbruch IK Paulson OB , et al. The Kety-Schmidt technique for repeated measurements of global cerebral blood flow and metabolism in the conscious rat. Acta Physiol Scand 1999; 165: 395–401. 10350234 10.1046/j.1365-201x.1999.00522.x

[143] Todd MM Weeks J. Comparative effects of propofol, pentobarbital, and isoflurane on cerebral blood flow and blood volume. J Neurosurg Anesthesiol 1996; 8: 296–303. 8884627 10.1097/00008506-199610000-00007

