# Supplementary material for: Stroke-prone salt-sensitive spontaneously hypertensive rats show higher susceptibility to spreading depolarization (SD) and altered hemodynamic responses to SD
Source: J Cereb Blood Flow Metab. 2022 Nov 3;43(2):210–30. doi: 10.1177/0271678X221135085 (PMC9903222; doi:10.1177/0271678X221135085)
Supplement: Supplementary material [file sj-pdf-1-jcb-10.1177_0271678X221135085.pdf]

Supplementary Material

Supplementary Materials and Methods

Electrical stimulation of SD

The dura was left intact and the stimulation site was covered with a thin film of mineral oil to reduce tissue drying. To lower the resistance between electrode and dura, electrode tips were dipped into conductive cream (SYNAPSE, Kustomer Kinetics, Arcadia, CA, USA). Stimulation pulses were generated using a programmable pulse generator (Master-8, AMPI Instruments, Jerusalem, Israel) connected to a mains-operated stimulus isolator (SIU-102, Warner Instruments, Hamden, CT, USA) to ensure a consistent compliance voltage of 100V. The stimulation protocol consisted of biphasic pulses of 150ms (+75ms/-75ms) duration with exponentially increasing intensities (340, 450, 600, 800, 1000, 1300, 1700, 2300, 3000, 4000, 5000, 6000, 7000, 8000, 9000, 10000mA) at 3-min intervals until SD was triggered.[1] SD threshold was defined as the lowest current necessary to trigger SD. In experiments in which SD could not be elicited even at maximum intensity, a pinprick was used to elicit SD. This was scored as maximum intensity (=10000mA).

Ex-vivo MRI

In series 1A, *ex-vivo* MRI scans were performed using the Aspect M2 MRI system (Aspect Imaging Technologies Ltd., Lod, Israel) as previously reported (**Figure 1(c)**).[2, 3] A T2-weighted (T2w) fast spin echo sequence (repetition time/echo time/number of excitations=3516/60/4, respectively; acquisition time=93.3 minutes) was performed and scans were collected with a 3cm field-of-view and data matrix of 256x256, resulting in a 0.12mm in-plane resolution and slice thickness of 0.7mm. Analysis was performed using in-house Matlab scripts. Images first underwent preprocessing to extract the brain volume of interest and the creation of 3D brain

objects and were then registered to a rat brain atlas (LONI Laboratory of Neuro Imaging Imaging, http://www.loni.usc.edu/atlases), enabling the automated segmentation into anatomical brain regions. Signal changes were measured in 5 brain regions (cortex, hippocampus, amygdala, diencephalon, and corpus callosum). For each region, the volume was measured and the percentage of voxels with hyperintense (abnormal) T2w signal (ABT2w%, indicating edema) was determined.[3] The Gaussian mixture model with three Gaussian probability density functions was applied to model the variety of intensities (**Figure 1(d)**). Voxels were clustered as a mixture of three Gaussians, namely: "low", white matter; "medium", mostly gray matter; and "high", ventricles or abnormally hyperintense regions. Hyperintensities were quantified by including voxels above a segmentation threshold, defined as intersection between the 2nd and 3rd Gaussian fit.

Experimental design and statistical analysis

No prior information was available which would have enabled us to perform sample size estimations based on evidence. We thus chose sample sizes which are standard in the field. The relatively large sample size used to test SD thresholds in series 1A and 1B compared with the sample size used to test SD-induced spreading ischemia in group 2 was based on our recent experience comparing SD thresholds between $\alpha^{2+/KOE4}$ mice and their wild-type littermates.[1] No animals/outliers were excluded from analysis. Within each individual series, the experiments were performed randomly. MRI data were automatically segmented and analyzed blindly. Statistical tests are given in the text and figure legends. If necessary, variables were submitted to logarithmic transformation to approach normal distribution. When appropriate and because ln(x+1), log(x+1), sqrt(x), or 1/x transformations or removal of outliers failed, we used two-way repeated measures analysis of variance (ANOVA) on ranks with post-hoc Bonferroni t-tests. Ranks are not normally distributed, but severe heteroscedasticity and outliers are tamed

by the ranking procedure, so violations of standard ANOVA assumptions are expected to be small. Differences were considered significant at $p \leq 0.05$. Data are shown as median and interquartile range (IQR), i.e. 25th and 75th percentile.

Pilot experiments in preparation for the present study were performed on two SHRsp on regular diet, one SHRsp on Japanese diet, four WKY on regular diet and one WKY on Japanese diet. Two SHRsp on regular diet and two SHRsp on Japanese diet but no WKY died during the experiment. Three SHRsp and three WKY on Japanese diet experienced technical problems during the experiment that precluded analysis. If BW before surgery or body temperature or MAP after surgery could be determined in these experiments, the values were included in the statistics.

Legends of Supplementary Videos

Supplementary Video 1. Brain topical application of NG-nitro-L-arginine (L-NNA) and elevated K^+ concentration in the artificial cerebrospinal fluid ($[K^+]_{aCSF}$) led to spontaneously recurring spreading depolarizations (SD) that induced spreading ischemias. SD induces tone alterations in resistance vessels, causing either predominant hyperperfusion followed by a mild oligemia (physiological hemodynamic response) in healthy tissue; or severe and prolonged initial hypoperfusion (inverse hemodynamic response = spreading ischemia) when the neurovascular unit is severely disturbed. Supplementary Video 1 shows the laser speckle contrast analysis (LASCA)-recorded perfusion changes of the first SD-induced spreading ischemia in a stroke-prone spontaneously hypertensive rat (SHRsp) on Japanese diet (experiment of series 2, compare **Figure 6(a)-6(c)**). The red and blue lines in the upper part of the movie show the perfusion changes in the respective regions of interest (ROI). The time axis is given in hours:minutes:seconds. Ag/AgCl-electrode: silver/silver chloride electrode, rCBF: regional cerebral blood flow compared to baseline (= 100%).

Supplementary Video 2. The normal hemodynamic response to spreading depolarization (SD) is characterized by a predominant hyperperfusion in naïve tissue. Supplementary Video 2 shows the laser speckle contrast analysis (LASCA)-recorded perfusion changes of a stimulus-induced SD accompanied by predominant hyperperfusion in a stroke-prone spontaneously hypertensive rat (SHRsp) on Japanese diet (experiment of series 3). The black line in the upper part of the movie shows the average change in perfusion units over the entire window, whereas the red and blue lines show the changes in the respective regions of interest (ROI). Comparison of the red, blue, and black lines in the top panel illustrates that the stimulation artifact is observed simultaneously throughout the window, while the SD-induced regional cerebral blood flow response to SD slowly propagates throughout the tissue. The time axis is given in hours:minutes:seconds. Ag/AgCl-electrode: silver/silver chloride electrode, rCBF: regional cerebral blood flow compared to baseline (= 100%).

Supplementary Table 1. Composition of the Japanese (methionine deficient, low potassium) diet

**Ingredient**	Unit	**Amount**
**Protein source (intact)**	**%**	——
Corn starch, pre-gelatinized	%	30.000
Sucrose	%	18.000
Maltodextrin, 10 DE	%	15.647
Cellulose powder	%	5.000
**Amino acid mixture**	**%**	**18.683 (total)**
• L-Lysine HCl	%	1.800
• DL-Methionine	%	0.003
• L-Cystine	%	0.350
• L-Threonine	%	0.900
• L-Tryptophan	%	0.200
• L-Arginine, free base	%	1.000
• L-Histidine HCl H_2O	%	0.500
• L-Valine	%	0.900
• L-Isoleucine	%	0.820
• L-Leucine	%	1.200
• L-Phenylalanine	%	0.750
• L-Tyrosine	%	0.500
• Glycine	%	2.510
• L-Glutamic acid	%	3.000
• L-Glutamine	%	2.000
• L-Aspartic acid	%	0.450
• L-Asparagine	%	0.600
• L-Proline	%	0.400
• L-Serine	%	0.400
• L- Alanine	%	0.400
Vitamin premixture	%	1.000
Mineral & trace element premixture	%	4.100
Choline Cl (50 %)	%	0.250
Sodium bicarbonate	%	0.300
Butylated hydroxytoluene	%	0.020
Soybean oil	%	7.000
Proximate contents		
Crude protein	%	**17.5**
Crude fat	%	7.1
Crude fibre	%	5.0

Crude ash	%	3.6
Carbohydrates	%	65.1
Lysine	%	1.40
Methionine	%	0.003
Methionine & Cystine	%	0.349
Threonine	%	0.88
Tryptophan	%	0.20
Calcium	%	0.65
Phosphorus	%	0.51
Magnesium	%	0.09
Sodium	%	0.20
Potassium	%	0.37
Energy (Atwater)	MJ/kg	16.5
• kcal/kg		3935
• kcal% Protein		18
• kcal% Fat		6
• kcal% Carbohydrates		66

1. Reiffurth C, Alam M, Zahedi-Khorasani M, et al. Na(+)/K(+)-ATPase alpha isoform deficiency results in distinct spreading depolarization phenotypes. *J Cereb Blood Flow Metab* 2020; 40: 622-638. 2019/03/02. DOI: 10.1177/0271678X19833757.
2. Lippmann K, Kamintsky L, Kim SY, et al. Epileptiform activity and spreading depolarization in the blood-brain barrier-disrupted peri-infarct hippocampus are associated with impaired GABAergic inhibition and synaptic plasticity. *J Cereb Blood Flow Metab* 2017; 37: 1803-1819. DOI: 10.1177/0271678X16652631.
3. Bar-Klein G, Lublinsky S, Kamintsky L, et al. Imaging blood-brain barrier dysfunction as a biomarker for epileptogenesis. *Brain* 2017; 140: 1692-1705. DOI: 10.1093/brain/awx073.
